# Circular RNA EIF4G3 suppresses gastric cancer progression through inhibition of β-catenin by promoting δ-catenin ubiquitin degradation and upregulating SIK1

**DOI:** 10.1186/s12943-022-01606-9

**Published:** 2022-07-02

**Authors:** Xueyan Zang, Jiajia Jiang, Jianmei Gu, Yanke Chen, Maoye Wang, Yu Zhang, Min Fu, Hui Shi, Hui Cai, Hui Qian, Wenrong Xu, Xu Zhang

**Affiliations:** 1 Aoyang Cancer Institute, Affiliated Aoyang Hospital of Jiangsu University, Zhangjiagang, 215600 Jiangsu China; 2 Jiangsu Key Laboratory of Medical Science and Laboratory Medicine, School of Medicine, Jiangsu University, Zhenjiang, 212013 Jiangsu China; 3 Department of Clinical Laboratory Medicine, Nantong Tumor Hospital, Nantong, 226361 Jiangsu China; 4 Key Laboratory of Molecular Diagnostics and Precision Medicine for Surgical Oncology in Gansu Province, Gansu Medical College of Jiangsu University, Lanzhou, 730000 Gansu China

**Keywords:** Circular RNA, Gastric cancer, δ-catenin, TRIM25, SIK1

## Abstract

**Background:**

Increasing studies suggest that circular RNAs (circRNAs) are critical regulators of cancer development and progression. However, the biological roles and mechanisms of circRNAs in gastric cancer (GC) remain largely unknown.

**Methods:**

We identified the differentially expressed circRNAs in GC by analyzing Gene Expression Omnibus (GEO) datasets. We explored the biological roles of circRNAs in GC by in vitro functional assays and in vivo animal studies. We performed tagged RNA affinity purification (TRAP), RNA immunoprecipitation (RIP), mass spectrometry (MS), RNA sequencing, luciferase reporter assays, and rescue experiments to investigate the mechanism of circRNAs in GC.

**Results:**

Downregulated expression of circular RNA EIF4G3 (circEIF4G3; hsa_circ_0007991) was found in GC and was associated with poor clinical outcomes. Overexpression of circEIF4G3 suppressed GC growth and metastasis through the inhibition of β-catenin signaling, whereas knockdown of circEIF4G3 showed the opposite effects. Mechanistic studies revealed that circEIF4G3 bound to δ-catenin protein to promote its TRIM25-mediated ubiquitin degradation and interacted with miR-4449 to upregulate SIK1 expression.

**Conclusion:**

Our findings uncovered a tumor suppressor function of circEIF4G3 in GC through the regulation of δ-catenin protein stability and miR-4449/SIK1 axis. CircEIF4G3 may act as a promising prognostic biomarker and therapeutic target for GC.

**Supplementary Information:**

The online version contains supplementary material available at 10.1186/s12943-022-01606-9.

## Introduction

Gastric cancer (GC) is the fifth most common cancer and the third leading cause of cancer-related death worldwide [1]. Although great improvement has been made, the early diagnosis rate, radical resection rate, and five year survival rate of GC patients are still unsatisfactory [2, 3]. Therefore, it is of urgent need to find more effective biomarkers and therapeutic targets for GC diagnosis and therapy.

Circular RNAs (circRNAs) are produced from precursor mRNA back-splicing and have been implicated as important regulators of gene expression [4–7]. CircRNAs were initially considered as byproducts of the biological process and though to have no functions. With the development of high-through sequencing and bioinformatics, circRNAs have been increasingly recognized as master regulators of various biological processes and key players in human health and diseases [8–10]. In particular, circRNAs have been shown to play important roles in cancer growth, metastasis, recurrence, and therapy resistance [11, 12]. Due to its closed structure and RNA exonuclease resistance, circRNAs are more stable than their linear counterparts, showing a potential to be used as cancer biomarkers [13].

Accumulating studies suggest that circRNAs participate in cancer biology via multiple mechanisms. For instance, ciRS-7/CDR1as (circular RNA sponge for miR-7) constitutes an competing endogenous RNA (ceRNA) network with miRNAs [12]. Interestingly, CDR1as interacts with IGF2BP3 and compromises its pro-metastatic functions [14]. CDR1as also interacts with p53 and blocks its degradation by MDM2 [15]. In addition to acting as miRNA sponges, circRNAs can interact with RNA binding proteins (RBPs), regulate RNA splicing and gene transcription, act as scaffold proteins, and translate into peptides [16–18]. For example, circRHOT1 promotes hepatocellular carcinoma (HCC) growth and metastasis by recruiting TIP60 to the NR2F6 promoter [19]. A novel protein cGGNBP2-184aa encoded by cGGNBP2 promotes intrahepatic cholangiocarcinoma (ICC) cell proliferation and metastasis [20]. Therefore, it deserves further study to reveal the multifaceted roles of circRNAs in the pathogenesis of GC and uncover the underlying molecular mechanisms.

In the present study, we demonstrated that a novel circRNA, hsa_circ_0007991 (named as circEIF4G3), was significantly downregulated in GC cells and tumor tissues of patients with GC. The decreased expression of circEIF4G3 was associated with disease progression and predicted an adverse overall survival. Functional studies indicated that circEIF4G3 overexpression suppressed the growth and metastasis of GC while circEIF4G3 knockdown showed an opposite effect. CircEIF4G3 bound to δ-catenin (catenin delta 1) protein and enhanced TRIM-25-mediated ubiquitination and degradation. CircEIF4G3 also acted as a miRNA sponge for miR-4449 and promoted the expression of its downstream target SIK1. Together, we identified circEIF4G3 as a tumor suppressive circRNA in GC, which may offer a new prognostic biomarker and therapeutic target for GC.

## Materials and methods

### Patients and clinical samples

A total of 103 paired tumor and adjacent non-tumor tissues from GC patients, 120 serum samples from GC patients, 50 serum samples from gastritis patients, and 120 serum samples from healthy donors were obtained from Nantong Tumor Hospital between April 2018 and September 2020. Specimens were collected in accordance with institutional protocols. Written informed consent was obtained from all the participants and the study was approved by Institutional Ethical Committee of Jiangsu University.

### Bioinformatic analysis of circRNA expression profile in Gene Expression Omnibus datasets

Microarray data was downloaded from the Gene Expression Omnibus (GEO) datasets and DESeq2 package was used to analyze differentially expressed circRNAs. Fold change ≥ 2 and P value < 0.05 were set as the threshold for significantly differential expression.

### Cell culture

Human gastric cancer cell lines (AGS, HGC-27, MKN-45 and SGC-7901) and HEK-293 T cell line were purchased from the Cell Bank of the Chinese Academy of Sciences (Shanghai, China). Human normal gastric mucosa epithelial cell line GSE-1 was obtained from Gefan Biological Technology (Shanghai, China). HEK-293 T and GSE-1 cells were cultured in high glucose-DMEM with 10% fetal bovine serum (FBS; Invitrogen, Shanghai, China). AGS cells were cultured in DMEM-F12 medium (Invitrogen) containing 10% FBS. HGC-27, MKN-45, and SGC-7901 cells were cultured in RPMI 1640 medium (Invitrogen) with 10% FBS. All the cells were cultured at 37℃ in a humidified 5% CO_2_ atmosphere.

### Plasmid and siRNA transfection

Specific targeting siRNAs and overexpressing plasmid were designed and synthesized by GenePharma (Shanghai, China) and Bersinbio (Guangzhou, China). A density of 2 × 10^5^/well cells were plated in 6-well plates and cultured until 50–70% confluent overnight. The plasmids and siRNAs were transfected into the cells with Lipofectamine 2000 (Life Technologies) in serum-free medium according to the manufacturer’s instructions. Cells were changed to complete medium at 6 h after transfection and cultured for another 30 h.

### Tagged RNA affinity purification (TRAP) assay

TRAP assay was used to determine the interaction between circRNA and proteins. Control and circEIF4G3 overexpressing vectors that contain the stem-loop structure of MS2 (MS2 and circRNA-MS2) and GST-MS2 overexpressing vector were constructed by Biosense (Guangzhou, China). MS2 and circRNA-MS2 vectors were co-transfected with GST-MS2 into GC cells to obtain the GST-MS2-circRNA complex. Then, the complex was pulled down by glutathione magnetic beads. The circRNA-binding proteins were identified by mass spectrometry and validated by western blot.

### Dual luciferase reporter assay

Cells were cultured in 24-well plates and transfected with control vector, miRNA-binding site containing wild type (WT) or mutant (MUT) vector, as well as predicted miRNA mimics or controls (GenePharma, Suzhou, China). After 48 h transfection, the luciferase activity was detected by the dual luciferase reporter assay system (Promega, MA, USA). The intensity of firefly luciferase was normalized to that of renilla luciferase. The fold change between each miRNA compared to NC was calculated.

### Immunohistochemistry (IHC)

For immunohistochemical analyses, 4% paraformaldehyde fixed tissues were embedded in paraffin and cut into 4 μm-thick sections. The sections were incubated with primary monoclonal antibody against Ki-67 (Cell Signaling Technology) followed by incubation with the secondary antibody for 30 min at room temperature. After being incubated with 3, 3’-Diaminobenzidine (3, 3’-DAB, Maxim, Fuzhou, China) for 5 min, the sections were counterstained with hematoxylin for 30 s. Finally, the sections were photographed under a TE2000 microscope (Nikon, Tokyo, Japan).

### RNA–protein immunoprecipitation (RIP)

RIP assays were performed by EZ-Magna RIP™ RNA-Binding Protein Immunoprecipitation Kit (Millipore, Billerica, MA, USA) according to the manufacturer’s instructions. Cells at approximately 90% confluence were incubated with complete RIP lysis buffer containing RNase inhibitor and protease inhibitor. Magnetic beads were pre-incubated with the anti-Ago2 antibody for 1 h at room temperature, and lysates were immunoprecipitated with beads at 4 °C overnight. The immunoprecipitated RNA complex were then purified and quantified by qRT-PCR. Normal rabbit IgG was used as the negative control.

### RNA sequencing

Total RNA were extracted from control and circEIF4G3 overexpressing GC cells and sent for sequencing by Illumina HiSeq sequencer (Cloundseq, Shanghai, China). Cutadapt, Hisat2, and Cuffdiff software were used to compare high-quality reads to the genome, obtain the FPKM value, and calculate the differentially expressed genes between control and circEIF4G3 overexpressing groups. The heatMap2 function in the R package was used for cluster analysis of differentially expressed mRNAs with FPKM values.

### LC–MS/MS

Proteins were subjected to digestion with the sequencing-grade trypsin. The samples were analyzed by liquid chromatography tandem mass spectrometry (LC-MS/MS) to obtain original mass spectrometry results. Byonic software was used to analyze the raw file and search the uniprot-Homo sapiens data to obtain the identified protein results.

### Co-immunoprecipitation (Co-IP) assay

To detect protein and protein interactions, cells were lysed by Pierce immunoprecipitation lysis buffer supplemented with a cocktail of proteinase inhibitors, phosphatase inhibitors and RNase inhibitor (Thermo, Waltham, MA). After incubation at 4 °C overnight, beads were washed with cell lysis buffer three times. The proteins were eluted from the magnetic beads for western blot analysis.

### In vivo* animal studies*

For xenograft tumor model, 4-week-old male BALB/c nude mice were purchased from the Model Animal Research Center at Nanjing University (Nanjing, China) and raised under controlled conditions with comfortable temperature and humidity. The mice were randomly divided into 2 groups (*n* = 5 for each group), subcutaneously injected with HGC-27 cells (5 × 10^6^ cells per mouse) that were transfected with circEIF4G3 overexpressing or control vectors. The tumor size was measured every week and calculated by using the flowing formula: Volume = width^2^ × length/2. The tumor tissues were harvested for hematoxylin and eosin (H&E) and IHC staining. The animal experiments were approved by the Animal Use and Care Committee of Jiangsu University.

### Statistical analysis

Statistical analyses were carried out by SPSS software (Chicago, IL, USA). Student’s *t*-test and *x*^2^-test was performed to analyze the significance of differences between groups. Survival analysis was plotted according to the Kaplan–Meier curves and log-rank test in GraphPad Prism 5. The correlations were analyzed using Pearson’s correlation coefficients. Differences were considered to be statistically significant at values of *P* < 0.05.

## Results

### CircEIF4G3 is downregulated in GC and its lower level predicts poor prognosis

**Fig. 1:**
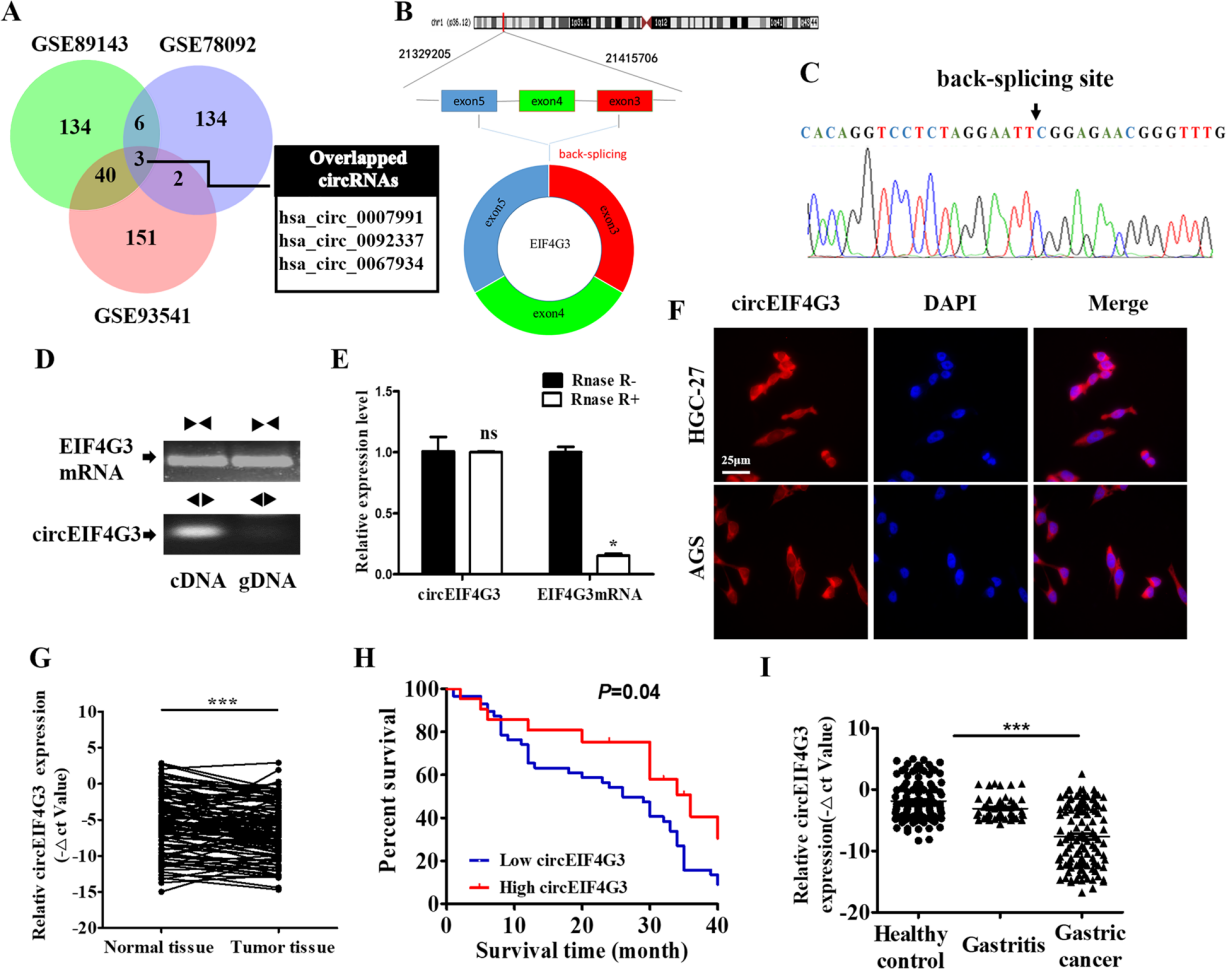
CircEIF4G3 is downregulated in GC. (A) GEO datasets were downloaded for integrated analyses of differentially expressed circRNAs. The common downregulated circRNAs were listed as indicated. (B) Genomic location of circEIF4G3. CircEIF4G3 was formed by the back-splicing of exons 3–5 of EIF4G3. (C) The backsplice junction site of circEIF4G3 was identified by Sanger sequencing. (D) PCR assay of gDNA and cDNA using divergent and convergent primers of circEIF4G3. (E) The stability of circEIF4G3 and EIF4G3 mRNA was detected by RNase R degradation assay. Data are shown as means ± SD (*n* = 3). (F) RNA FISH analysis for circEIF4G3 in GC cells. Scale bar = 25 μm. (G) CircEIF4G3 expression levels in tumor tissues and adjacent non-tumor tissues of patients with GC were detected by qRT-PCR (*n* = 103). (H) The association between circEIF4G3 expression level and overall survival time was analyzed by Kaplan–Meier plot. Log-rank tests were used to determine statistical significance. (I) CircEIF4G3 expression levels in serum of GC patients, gastritis and healthy controls were detected by qRT-PCR. *P < 0.05; ***P < 0.001

To identify the differentially expressed circRNAs in GC, we analyzed microarray datasets (GSE89143, GSE78092, and GSE93541) from Gene Expression Omnibus (GEO, https://www.ncbi.nlm.nih.gov/geo/). We found that several common circRNAs were differentially expressed between tumor tissues and adjacent non-tumor tissues in these datasets (Fig. 1A, Supplementary Fig. 1A). Considering the relative expression level and detection specificity, we chose hsa_circ_0007991 as the target for next study. The information of hsa_circ_0007991 can be queried in both Circbank and Circbase. Hsa_circ_0007991 was composed of exons 3–5 of the linear transcript of EIF4G3 gene with a length of 301 nucleotides (abbreviated as circEIF4G3) (Fig. 1B). Sequencing results confirmed the existence of back-splicing site in divergent primers-amplified PCR product (Fig. 1C). In accordance, circEIF4G3 was validated by PCR amplification using divergent primers from cDNA but not gDNA of GC cells (Fig. 1D). Endogenous circEIF4G3 was resistant to RNase R digestion, while linear EIF4G3 mRNA was notably reduced by RNase R treatment (Fig. 1E). RNA-FISH assay indicated that circEIF4G3 mainly located in the cytoplasm of GC cells (Fig. 1F). Subcellular fractionation assay also showed the same results (Supplementary Fig. 1B).

We next examined the expression of circEIF4G3 in human GC cells and tissues by qRT-PCR. The result showed that circEIF4G3 expression levels were decreased in GC cells, including HGC-27, MKN-45, AGS, NCI-N87, and SGC-7901, as compared to the normal human gastric mucosal epithelial cell line (GES-1) (Supplementary Fig. 1C). We then verified the expression of circEIF4G3 in paired tumor and non-tumor tissue samples from patients with GC. We observed that the expression of circEIF4G3 significantly decreased in tumor tissues compared to adjacent non-tumor tissues (Fig. 1G). Further, we evaluated the association between circEIF4G3 expression level and the pathological parameters. As shown in Additional Table 1, circEIF4G3 expression levels were negatively associated with TNM stage and venous invasion while showed no significant association with genders, ages, tumor sizes, and differentiation stages. The lower expression of circEIF4G3 was strongly associated with a shorter survival time of patients with GC (Fig. 1H). Recently, several studies demonstrate that deregulated circRNAs originating from tumor tissues are stable and easily detected in the serum or plasma of cancer patients. We found that the expression of circEIF4G3 was much lower in the serum of GC patients than those of healthy individuals (Fig. 1I). The receiver operating characteristic (ROC) curve was used to investigate the diagnostic value of circEIF4G3 in serum as a biomarker for GC. Serum circEIF4G3 distinguished GC cases from healthy controls with AUC of 0.797. The sensitivity and specificity of circEIF4G3 for the diagnosis of GC were 0.59 and 0.98, respectively (Supplementary Fig. 1D). As indicated in Additional Table 2, we found that serum circEIF4G3 expression levels were inversely associated with lymph node and distant metastasis. Together, these data suggests that circEIF4G3 is downregulated in GC and may serve as a prognostic biomarker.

### CircEIF4G3 overexpression attenuates GC growth and metastasis

**Fig. 2:**
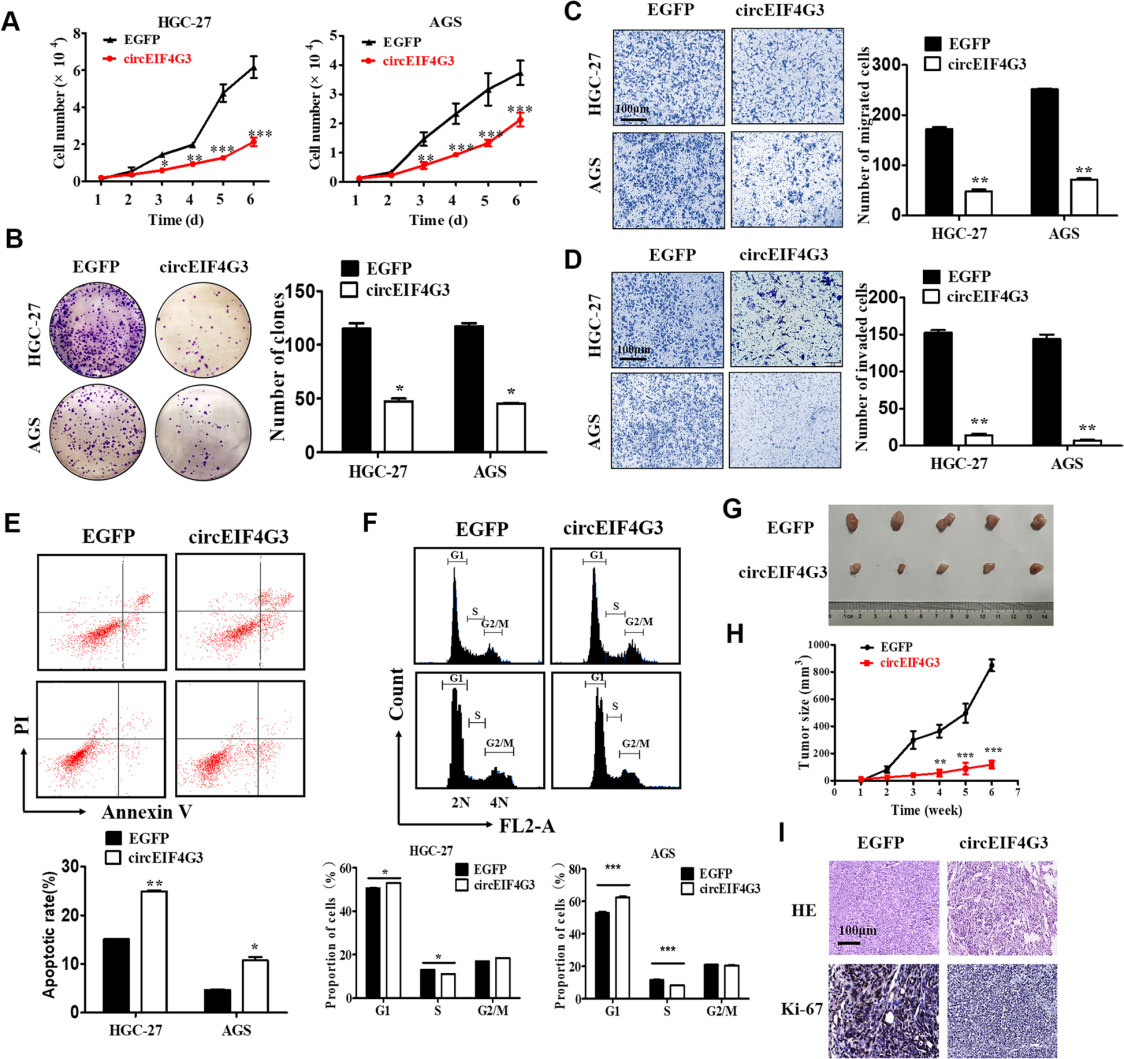
CircEIF4G3 overexpression attenuates GC growth and metastasis. (A) Cell counting assays for control and circEIF4G3 overexpressing GC cells. (B) The impact of circEIF4G3 overexpression on GC cell proliferation was determined by cell colony formation assay. (C-D) Transwell migration (C) and matrigel invasion assays (D) for control and circEIF4G3 overexpressing GC cells. (E–F) Flow cytometry analyses of cell apoptosis and cell cycle distribution in control and circEIF4G3 overexpressing groups. Data are shown as means ± SD (*n* = 3). (G-H) The volume and weight of subcutaneous xenograft tumors from mice injected with control and circEIF4G3 overexpressing GC cells (*n* = 5 mice/group). (I) HE staining and immunohistochemical staining of Ki-67 in mouse xenograft tumors with or without circEIF4G3 overexpression. *P < 0.05, **P < 0.01, ***P < 0.001; Scale bar = 100 μm

To further explore the biological roles of circEIF4G3, we performed gain-of- and loss-of-function studies (Supplementary Fig. 2A and 3A). The results of cell growth and colony formation assays showed that ectopic expression of circEIF4G3 inhibited GC cell proliferation (Fig. 2A and 2B). CircEIF4G3 overexpression dramatically suppressed the migration and invasion of cells (Fig. 2C and 2D). Flow cytometry results showed that circEIF4G3 overexpression caused an increase in the percentage of apoptotic cells (Fig. 2E) and a dramatic reduction in S-phase and increase in G1 phase of HGC-27 and AGS cells (Fig. 2F). The results of qRT-PCR and western blot showed that the mRNA and protein levels of E-cadherin increased while that of N-cadherin, Vimentin and slug decreased in circEIF4G3 overexpressing cells compared to control cells (Supplementary Fig. 2B and 2C).

We then established a mouse xenograft tumor model to validate the effect of circEIF4G3 on GC growth. We injected circEIF4G3 overexpressing and control HGC-27 cells into nude mice and monitored tumor growth regularly. The results showed that circEIF4G3 overexpression significantly inhibited tumor growth (Fig. 2G). After 6 weeks, we sacrificed the mice and calculated the tumor weights. Similarly, circEIF4G3 overexpression led to smaller tumor sizes (Fig. 2H). Immunohistochemical staining results revealed that the percentage of Ki-67-positive proliferating cells decreased in circEIF4G3 overexpressing group compared to control group (Fig. 2I).

Subsequently, we designed two siRNAs specifically targeting the backsplicing site of circEIF4G3 in GC cells (Supplementary Fig. 3A and 3B). As shown in Supplementary Fig. 3C-F, circEIF4G3 knockdown promoted GC cell proliferation, migration and invasion. The mRNA and protein levels of N-cadherin, Vimentin and slug were increased in circEIF4G3 knockdown group (Supplementary Fig. 3G-H). Flow cytometry results showed that circEIF4G3 knockdown decreased the percentage of apoptotic cells (Supplementary Fig. 3I) and induced an increase in S phase of GC cells (Supplementary Fig. 3 J). Taken together, these results indicate that circEIF4G3 performs tumor suppressive roles in GC.

### CircEIF4G3 destabilizes δ-catenin protein and inactivates β-catenin signaling in GC cells

**Fig. 3:**
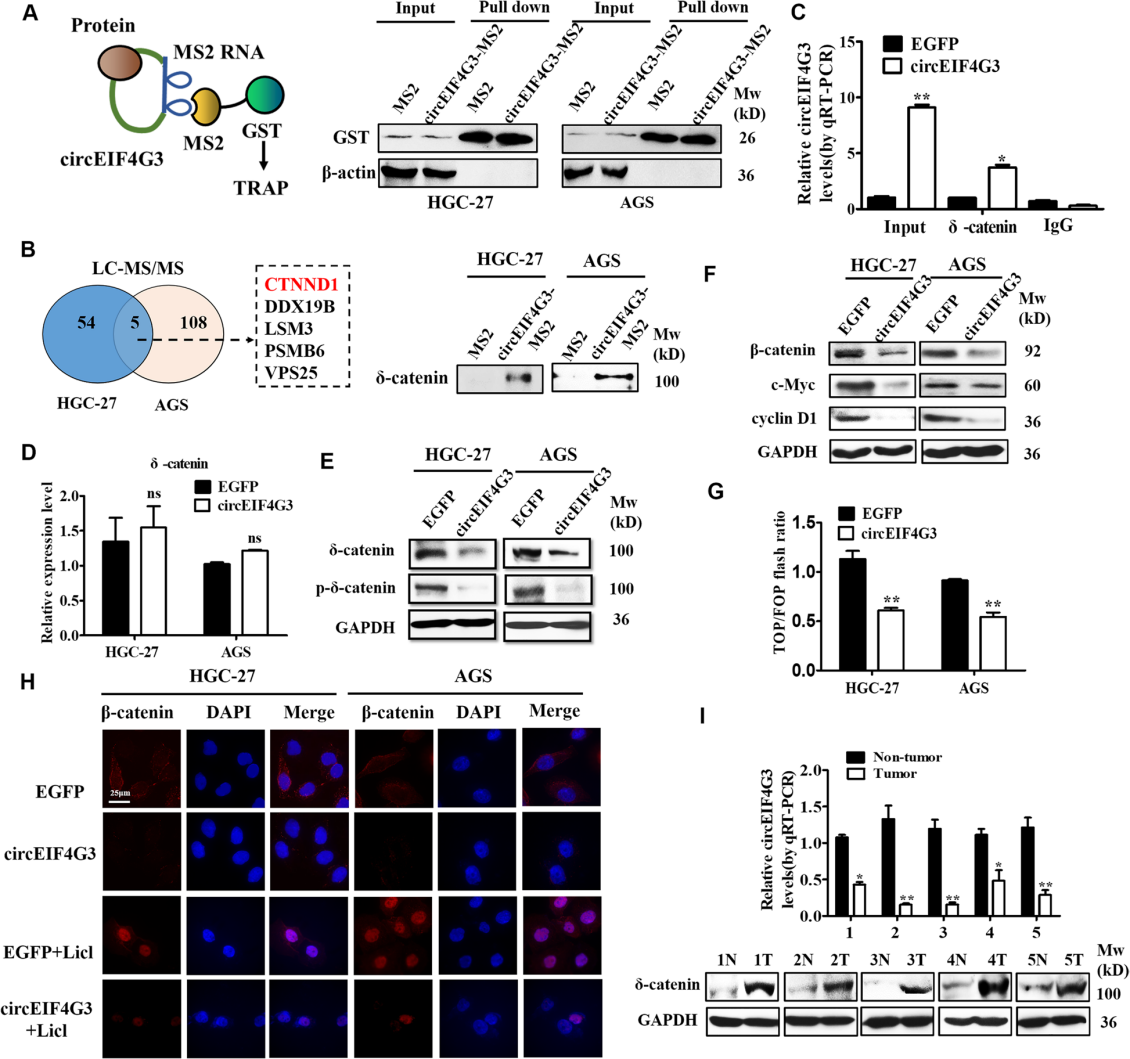
CircEIF4G3 destabilizes δ-catenin protein and inactivates β-catenin signaling. (A) TRAP experiment and LC-MS/MS analysis. GST protein expression was detected by western blot. δ-catenin protein (encoded by CTNND1 gene) was identified and indicated. (B) The binding of circEIF4G3 to δ-catenin protein was validated by TRAP assay followed by western blot. (C) The association of circEIF4G3 to δ-catenin protein was determined by RIP assay followed by qRT-PCR. (D) The relative expression levels of δ-catenin gene in GC cells with or without circEIF4G3 overexpression. (E–F) Western blot analyses of the indicated proteins in circEIF4G3 overexpressing GC cells. (G) TOP/FOP flash luciferase reporter assays for β-catenin activity in control and circEIF4G3 overexpressing GC cells. Results were normalized to the Renilla internal control. (H) Immunofluorescent staining of β-catenin (red) in control and circEIF4G3 overexpressing GC cells with or without LiCl treatment. Scale bar = 25 μm. (I) QRT-PCR analysis of circEIF4G3 expression and western blot assay for δ-catenin protein expression in paired tumor and non-tumor tissues. Data are shown as means ± SD. *P < 0.05, **P < 0.01, ***P < 0.001

To test whether circEIF4G3 exerts its function via interacting with proteins, we conducted tagged RNA affinity purification (TRAP) assay and mass spectrometry analyses to detect the specific proteins bound by circEIF4G3. The results of LC-MS/MS revealed that several proteins were consistently pulled down by circEIF4G3 in two GC cell lines (Fig. 3A). Five potential circEIF4G3-interacting proteins were identified through comprehensive analysis (Fig. 3B). We focused on δ-catenin as it has been well recognized as a key play in the progression of many human cancers [21]. We then utilized TRAP and western blot to verify the interaction between circEIF4G3 and δ-catenin protein (Fig. 3B). Meanwhile, RIP assay results also indicated that circEIF4G3 was enriched in RNA co-precipitated by anti-δ-catenin antibody in GC cells (Fig. 3C). Intriguingly, circEIF4G3 overexpression did not affect δ-catenin mRNA level but reduced its protein level in GC cells (Fig. 3D-E). δ-catenin is an important modulator of the canonical β-catenin signaling [22]. We found that circEIF4G3 overexpression dramatically decreased β-catenin protein level in GC cells while silencing circEIF4G3 had an opposite effect (Fig. 3F, Supplementary Fig. 3 K). β-catenin regulates various downstream targets including cyclin D1 and c-Myc to promote tumor progression [23]. We observed that circEIF4G3 overexpression inhibited while circEIF4G3 knockdown promoted the expression of c-Myc and cyclin D1 in GC cells (Fig. 3F, Supplementary Fig. 3 K). Furthermore, the luciferase reporter activity of β-catenin was reduced in circEIF4G3 overexpressing group compared to control group (Fig. 3G). To further verify the role of circEIF4G3 in regulating β-catenin signaling, we used the β-catenin pathway activator LiCl. Compared with control group, Licl treatment induced the nucleus translocation of β-catenin in GC cells, while circEIF4G3 overexpression remarkably suppressed this effect (Fig. 3H). Consistent with the in vitro results, the expression of δ-catenin protein was elevated in tumor tissues of patients with GC who had low levels of circEIF4G3 (Fig. 3I, Supplementary Fig. 5F). Moreover, δ-catenin expression was also decreased in mouse tumor tissues in circEIF4G3 overexpressing group (Supplementary Fig. 8).

Next, we confirmed that δ-catenin exerted oncogenic activities in GC cells as δ-catenin overexpression notably increased the proliferation, migration, and invasion abilities of GC cells (Supplementary Fig. 4A-D). We further performed rescue experiments and demonstrated that δ-catenin overexpression, at least partially, reversed the effects of circEIF4G3 on suppressing GC cell proliferation (Supplementary Fig. 5A-B), migration, and invasion (Supplementary Fig. 5C-D). In addition, δ-catenin overexpression also partially abrogated the decrease of β-catenin, c-Myc, and cyclin D1 expression by circEIF4G3 overexpression (Supplementary Fig. 5E). In summary, these data suggests that circEIF4G3 regulates β-catenin signaling by interacting with δ-catenin.

### CircEIF4G3 promotes TRIM25-mediated ubiquitin degradation of δ-catenin

**Fig. 4:**
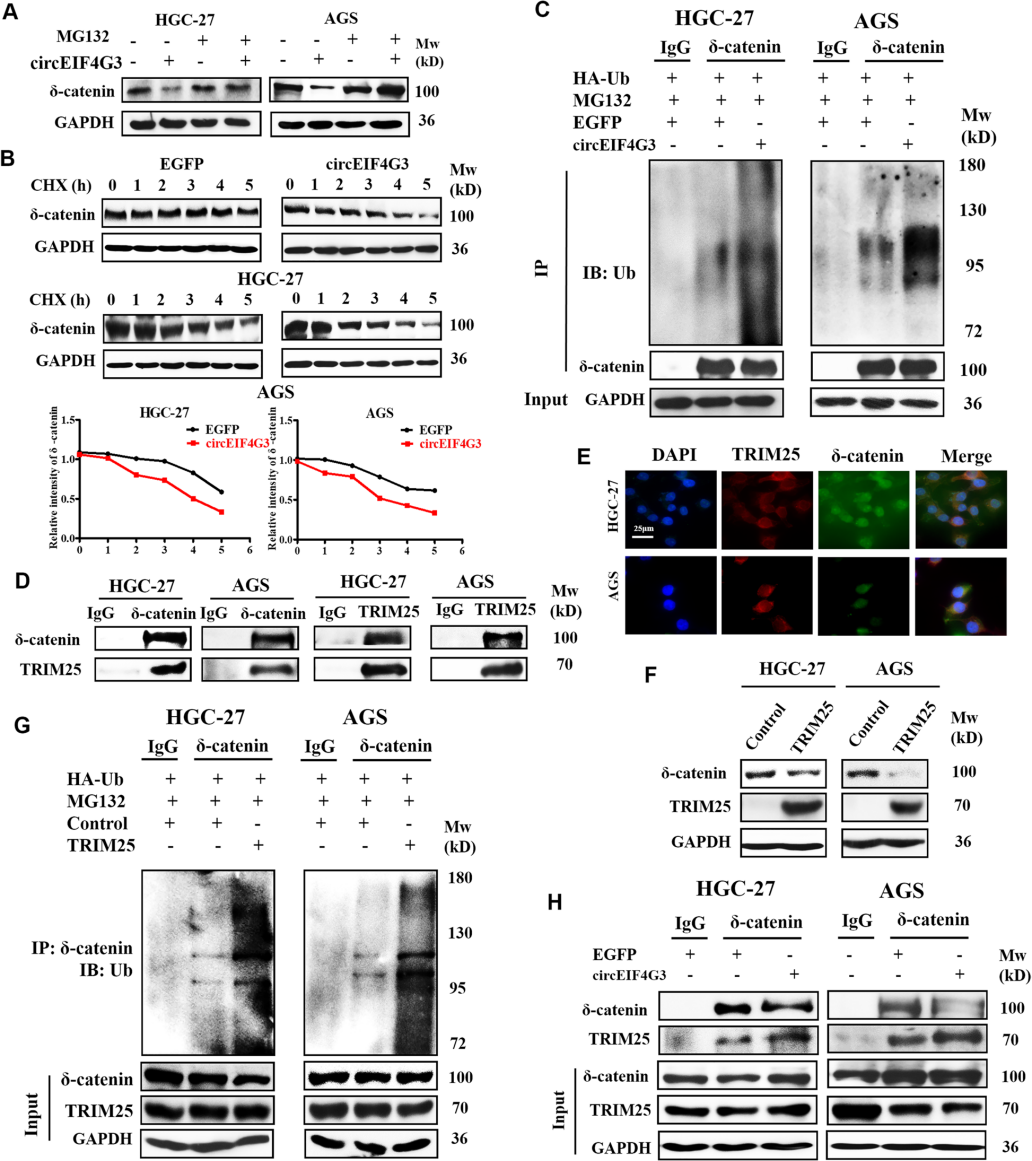
CircEIF4G3 acts as a scaffold to promote δ-catenin ubiquitin degradation by TRIM25. (A) The expression of δ-catenin protein in GC cells with or without circEIF4G3 overexpression after treatment with MG-132 (40 μM) for 6 h was determined by western blot. (B) Protein biosynthesis in GC cells was blocked with cycloheximide (CHX). The protein levels of δ-catenin in GC cells of indicated groups were determined at indicated time points by western blot. The corresponding quantification curve was exhibited. (C) GC cells were transfected with ubiquitin (Ub) and circEIF4G3 overexpressing plasmid and treated with MG-132. The ubiquitination of δ-catenin was determined by immunoprecipitation (IP) with δ-catenin antibody followed by Western blot with ubiquitin antibody. (D) The binding of TRIM25 and δ-catenin in GC cells was detected by Co-IP. (E) Immunofluorescent staining for the co-localization of δ-catenin protein (green) and TRIM25 protein (red) in GC cells. Scale bar = 25 μm. (F) δ-catenin protein levels in control and TRIM25 overexpressing GC cells were examined by western blot. (G) The ubiquitination of δ-catenin in GC cells with TRIM25 overexpression. (H) GC cells were transfected with circEIF4G3 overexpressing plasmid and the binding of TRIM25 to δ-catenin was determined by Co-IP and western blot

Considering that circEIF4G3 alters δ-catenin protein but not mRNA level (Fig. 3D-E), we speculated that circEIF4G3 may destabilize δ-catenin protein by ubiquitination/degradation system. To this end, we used a proteasome inhibitor MG132 to explore the effect of circEIF4G3 on δ-catenin protein degradation. As shown in Fig. 4A, the reduction of δ-catenin protein by circEIF4G3 overexpression was restored by MG132. We then transfected GC cells with circEIF4G3 and monitored the half-life of δ-catenin protein after CHX treatment. Compared to control group, circEIF4G3 overexpression evidently promoted δ-catenin protein degradation, thus shortening its half-life (Fig. 4B). Bioinformatics analysis results showed that δ-catenin protein has multiple ubiquitination modification sites. The levels of ubiquitinated δ-catenin protein were increased in GC cells when circEIF4G3 was overexpressed in the presence of ubiquitin (Fig. 4C). These results suggest that circEIF4G3 regulates δ-catenin protein stability via enhancing its ubiquitination-dependent degradation.

We screened the proteins co-precipitated by circEIF4G3 in TRAP assay for potential E3 ligase. Western blot result showed that TRIM25 was detectable in the proteins co-precipitated by circEIF4G3 (Supplementary Fig. 6A). On the contrary, β-TrCP, a previously reported E3 ligase for δ-catenin ubiquitination, was not found in the proteins co-precipitated by circEIF4G3. Previous studies demonstrate that TRIM family proteins, including TRIM25, promote the degradation of their substrates by the ubiquitin proteasome pathway [24, 25]. We then performed co-immunoprecipitation (Co-IP) assay and the results showed that TRIM25 bound to δ-catenin (Fig. 4D). RNA FISH and immunofluorescence results showed that circEIF4G3, TRIM25, and δ-catenin co-localized in the cytoplasm of GC cells (Fig. 4E, Supplementary Fig. 6B). More importantly, we found that TRIM25 overexpression decreased the protein levels but not mRNA levels of δ-catenin (Fig. 4F, Supplementary Fig. 6C). The ubiquitination of δ-catenin were increased in GC cells with TRIM25 overexpression (Fig. 4G). Taken together, these data suggest that TRIM25 functions as a ubiquitin E3 ligase for circEIF4G3-regulated δ-catenin ubiquitination and degradation in GC cells.

Previous studies suggest that TRIM25 uses RNA as a scaffold for efficient ubiquitination of its targets [26]. We then explored whether the ubiquitin-ligase activity of TRIM25 for δ-catenin is dependent on the presence of circEIF4G3. As expected, the loss of circEIF4G3 notably reduced TRIM25-meidated ubiquitination and degradation of δ-catenin in GC cells (Supplementary Fig. 6D). We performed Co-IP assay to further explore whether circEIF4G3 acts as a scaffold to enhance the binding of TRIM25 with δ-catenin and found that the association between TRIM25 and δ-catenin was enhanced in GC cells by circEIF4G3 overexpression (Fig. 4H). These results indicate that circEIF4G3 acts as a scaffold to promote the interaction between TRIM25 and δ-catenin and subsequently facilitates TRIM25-mediated ubiquitination and degradation of δ-catenin.

### CircEIF4G3 acts as a miR-4449 sponge in GC

**Fig. 5:**
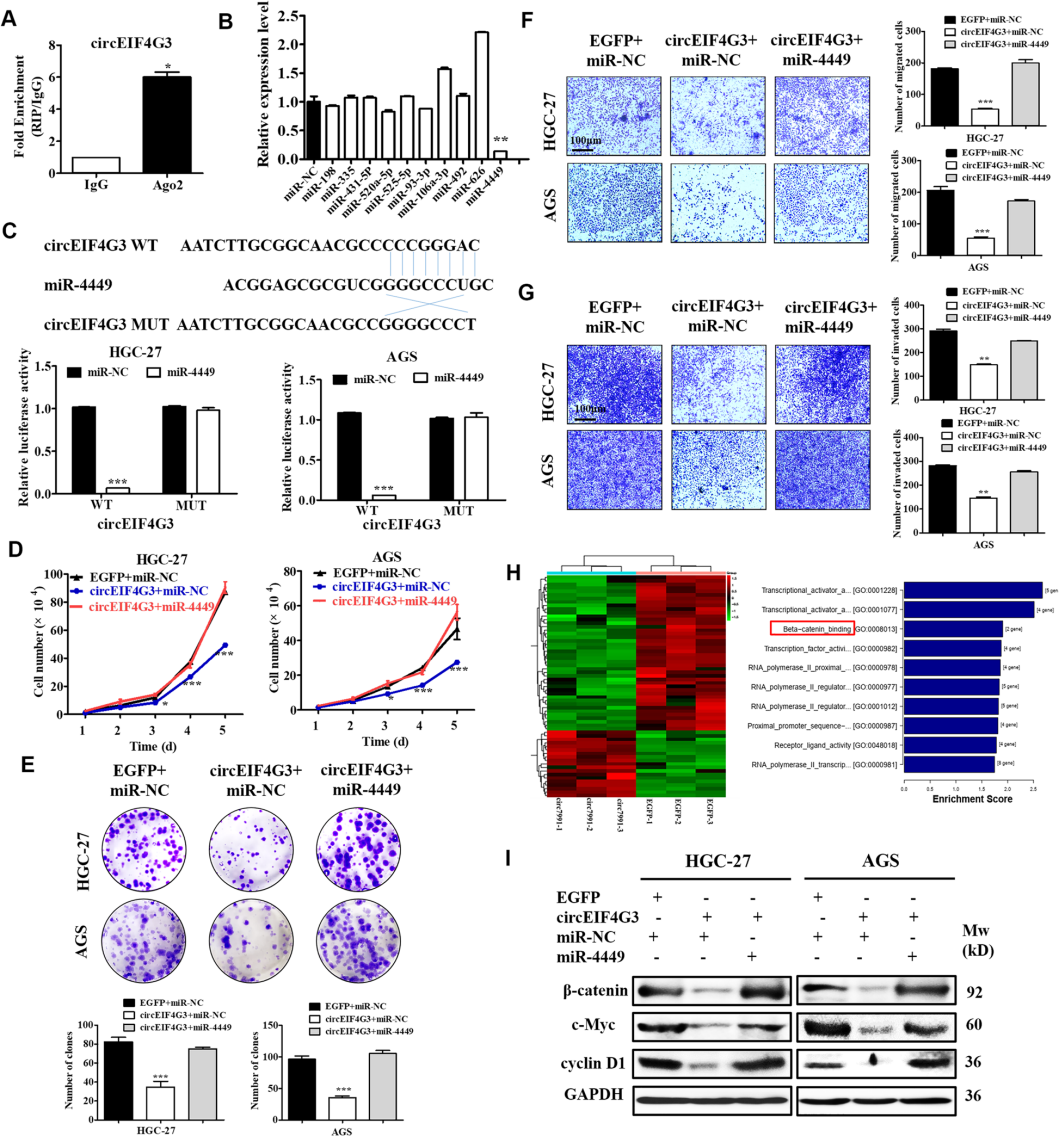
CircEIF4G3 functions as a miR-4449 sponge. (A) RIP assay was applied using Ago2 antibody in GC cells. The relative RNA level of circEIF4G3 was detected by qRT-PCR. (B) HEK293T cells were transfected with different plasmid or miRNAs as indicated. Relative luciferase activity was detected and normalized to the Renilla internal control. (C) Luciferase reporter assay for luciferase activity of circEIF4G3 in GC cells co-transfected with miRNA mimics. (D-G) Rescue experiments for cell growth curve (D), colony formation (E), transwell migration (F), and matrigel invasion assays (G) in GC cells co-transfected with circEIF4G3 and miR-4449. Data are shown as means ± SD (*n* = 3). Scale bars = 100 μm. (H) GO analysis and enriched GO terms of differentially expressed genes in GC on their biological process (BP). (I) The expression of indicated proteins in rescue experiment was determined by western blot. *P < 0.01, **P < 0.01, ***P < 0.001

Previous studies suggest that circRNAs regulate target gene expression by sponging miRNAs [27–29]. We then examined whether circEIF4G3 could function as a miRNA sponge. RIP assay results showed that circEIF4G3 was specifically enriched in beads containing Ago2 antibody compared with control IgG, suggesting the occupancy of Ago2 in the region of circEIF4G3 (Fig. 5A). We analyzed the potential targeted miRNAs of circEIF4G3 through bioinformatic methods (STARBASE, version 2.0 and circbank). We designed a luciferase screening assay using circEIF4G3-luciferase reporter and miRNA mimics and found that the luciferase activity was notably reduced when co-transfected with miR-4449 (Fig. 5B). We further identified a potential binding site in circEIF4G3 for miR-4449 (Fig. 5C). Further analysis showed that miR-4449 mimics notably suppressed the luciferase activity of circEIF4G3 wild-type reporter while not affected that of circEIF4G3 mutant reporter (Fig. 5C). We further confirmed that miR-4449 overexpression enhanced GC cell proliferation, migration, and invasion while circEIF4G3 overexpression antagonized these effects (Fig. 5D-G), indicating that circEIF4G3 may partially exert its tumor suppressive effect by sponging miR-4449 in GC.

To identify the downstream signaling pathway and target genes that may be regulated by circEIF4G3, we performed RNA-seq for control and circEIF4G3 overexpressing GC cells. Pathway enrichment analyses showed that the altered transcripts by circEIF4G3 overexpression were enriched in many signaling pathways associated with tumor progression, including the β-catenin signaling (Fig. 5H). Therefore, we next explored whether circEIF4G3 could regulate β-catenin signaling through miR-4449 in GC cells. The results showed that circEIF4G3 overexpression decreased the protein levels of β-catenin, c-Myc, and cyclin D1, while simultaneous overexpression of miR-4449 mimics compromised this effect (Fig. 5I), indicating that circEIF4G3 may also inhibit β-catenin signaling by interacting with miR-4449.

### CircEIF4G3 regulates miR-4449/SIK1 axis to inactivate β-catenin signaling

**Fig. 6:**
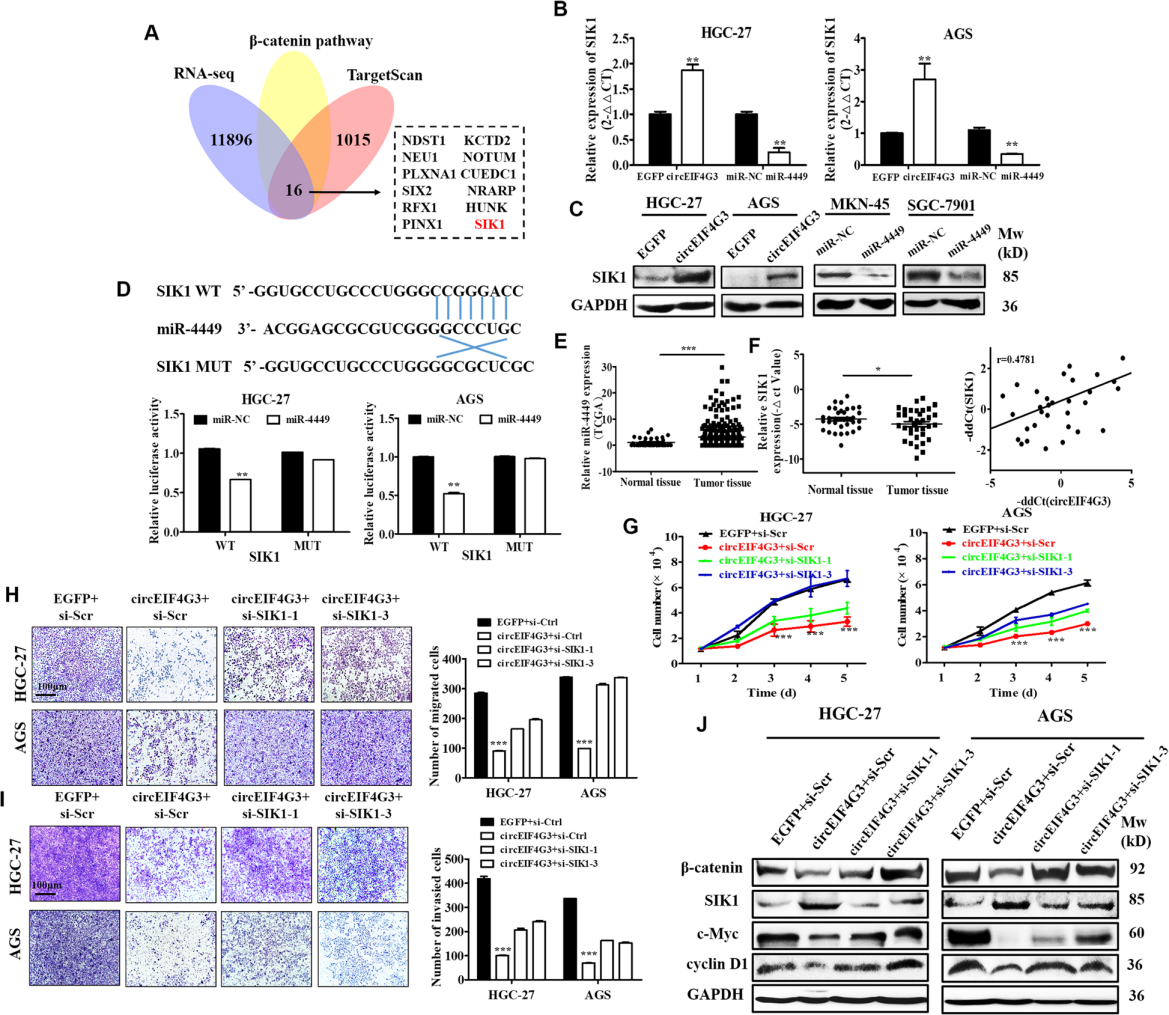
SIK1 is a target of miR-4449 and circEIF4G3 modulates miR-4449/SIK1 axis in GC. (A) Bioinformatic analysis of potential target genes for miR-4449. (B-C) The mRNA expression (B) and protein levels (C) of potential target genes for miR-4449 in GC cells transfected with circEIF4G3 or miRNA mimics was determined by qRT-PCR and Western blot. (D) Relative luciferase activities of wild-type (WT) and mutated (MUT) SIK1 reporter plasmid in GC cells co-transfected with miR-4449 mimics. (E) TCGA data analysis for the expression of miR-4449 in GC tissues. (F) QRT-PCR analysis for the expression of SIK1 in GC tissues. Correlation analysis of circEIF4G3 and SIK1 gene expression in tumor tissues of patients with GC. CircEIF4G3 and si-SIK1 were co-transfected into GC cells. GC cell proliferation (G), migration (H) and invasion (I) were determined. (J) The expression of β-catenin, c-Myc, and cyclin D1 was determined by western blot. Scale bar = 100 μm. Data are shown as means ± SD. *P < 0.01, **P < 0.01, ***P < 0.001

We further investigated the target genes of miR-4449 that are regulated by circEIF4G3. RNA-seq results combined with bioinformatic prediction using TargetScan and miRDB identified several target genes (Fig. 6A). We chose salt-inducible kinases (SIK1) for further study as SIK1 mRNA and protein levels were upregulated in circEIF4G3 overexpressing and miR-4449 inhibitor groups while they decreased in circEIF4G3 knockdown and miR-4449 mimics groups, respectively (Fig. 6B-C, Supplementary Fig. 7A-B). The results of dual-luciferase reporter assay showed that miR-4449 mimics reduced the luciferase activity of reporter genes containing SIK1 binding site for miR-4449 when compared with control group, and the reduction was abrogated when the binding site in SIK1 for miR-4449 was mutated (Fig. 6D). TCGA data analysis showed that miR-4449 was up-regulated in tumor tissues compared to non-tumor tissues of patients with GC (Fig. 6E). We further investigated SIK1 gene expression in 36 paired tumor and adjacent non-tumor tissues and found that the expression of SIK1 was downregualted in GC and positively associated with that of circEIF4G3 (Fig. 6F, Supplementary Fig. 7C). Moreover, SIK1 expression was also increased in mouse tumor tissues in circEIF4G3 overexpressing group (Supplementary Fig. 8).

SIK1 has been reported as a tumor suppressor gene in hepatocellular carcinoma by regulating β-catenin signaling [30]. Thus, we explored whether circEIF4G3 modulates β-catenin signaling through SIK1 in GC. Our results showed that SIK1 overexpression markedly decreased β-catenin, c-Myc, and cyclin D1 protein levels, as well as the luciferase activity of β-catenin in GC cells (Supplementary Fig. 7D-E). The effect of SIK1 on the proliferation and invasion of GC cells was also examined. As shown in Supplementary Fig. 7F-H, SIK1 overexpression resulted in a strong inhibition of GC cell proliferation, migration and invasion. Then, we overexpressed circEIF4G3 and knocked down SIK1 in GC cells simultaneously (Supplementary Fig. 7I). Our results revealed that GC cell proliferation, migration and invasion were greatly inhibited by circEIF4G3 overexpression; however, this inhibitory effect was reversed by simultaneous knockdown of SIK1 (Fig. 6G-I). The similar effect was also observed in the expression and transactivity of β-catenin (Fig. 6J). Taken together, these results indicate that SIK1 is a direct target of miR-4449 and circEIF4G3 regulates miR-4449/SIK1 axis to inactivate β-catenin signaling in GC.

## Discussion

The regulatory potential of circRNAs in gene expression has become a focus in cancer biology [31]. Increasing evidence suggests that circRNAs are aberrantly expressed in multiple cancers [32], including lung cancer [33], breast cancer [34], colorectal cancer [35], and hepatocellular carcinoma [36]. CircRNAs can be utilized as promising biomarkers for cancer diagnosis and prognosis due to their high stability and specific loop structure [37]. In this study, we identified circEIF4G3, a novel circRNA that was produced by backsplicing of EIF4G3 gene transcript, was downregulated in tumor tissues and serum of patients with GC. We found that the patients with GC who had a high level of circEIF4G3 presented significantly better survival than those who had a low level, which provides a new prognostic biomarker for GC. Moreover, the gain-of- and loss-of-function studies suggest that circEIF4G3 overexpression suppressed while its knockdown promoted cancer progression, indicating that circEIF4G3 plays a tumor suppressive role in GC.

FISH assays showed that circEIF4G3 was mainly distributed in the cytoplasm, where circRNAs may function as a miRNA sponge, interact with RNA binding proteins (RBPs) [38], or encode proteins [39, 40]. Emerging studies suggest that circRNAs may play important roles in cancer progression by interacting with RBPs. For example, circRNAs derived from HUR restrains CNBP-facilitated HUR expression, resulting in the repression of GC progression [41]. CircZKSCAN1 binds to FMRP and blocks the interaction between FMRP and CCAR1 in HCC cells, subsequently inhibiting the transactivity of Wnt signaling pathway [42]. We searched the circRNADb database and found no open reading frame (ORF) in the sequence of circEIF4G3, suggesting that the probability of encoding protein by circEIF4G3 is low. We then developed a highly specific circRNA pulldown assay and identified the potential interacting proteins of circEIF4G3 by mass spectrometry. We validated that circEIF4G3 directly bound to δ-catenin and promoted its degradation by facilitating the interaction between δ-catenin and TRIM25. δ-catenin, also named as p120-catenin, is a member of an emerging subfamily of Armadillo repeats (ARMs) proteins [43] and a regulator of β-catenin signaling [22]. δ-catenin is a multifaceted intracellular signaling protein, which may serve as an oncogene through driving migration and anchorage independence [44–46]. Due to the epithelial to mesenchymal transition, the loss of E-cadherin function or expression during cancer progression leads to the transfer of δ-catenin from the cell membrane to the cytoplasm or nucleus [47, 48]. δ-catenin modulates the canonical β-catenin signaling by forming a complex with its specific binding partner Kaiso [22]. Emerging evidence suggests that δ-catenin plays an important role in the development and progression of cancers [47, 49, 50]. Tang et al. demonstrate that δ-catenin regulates EMT, HCC cell invasion, and metastasis through the activation of β-catenin signaling pathway [49]. However, little is known about the regulation of δ-catenin in cancer. Herein, we found that δ-catenin promoted GC cell growth and metastasis. Moreover, circEIF4G3 could facilitate TRIM25-mediated ubiquitin degradation of δ-catenin. TRIM25 has been previously reported to be bound by non-coding RNAs to exert its E3 ubiquitin ligase activity and regulate antiviral innate immunity [51]. A more recent study also demonstrates that TRIM25 promotes HCC cell survival and growth through targeting Keap1-Nrf2 pathway [52]. Co-IP results showed that TRIM25 is the E3 ubiquitin ligase that interacts with δ-catenin and degrades it via the ubiquitin–proteasome pathway in GC cells. We also found that circEIF4G3 bound to δ-catenin and promoted its association with TRIM25, leading to increased ubiquitination of δ-catenin by TRIM25. These data provides a new mechanism for the regulation of δ-catenin protein stability by circRNAs.

Since most circRNAs contain miRNA response elements (MREs), they can also serve as miRNA sponges [12]. For example, circTP63 has conserved binding sites for miR-873-3p and promotes lung squamous cell carcinoma progression by upregulating FOXM1 [53]. Circ-RanGAP1 acts as a competing endogenous RNA for miR-877-3p to increase VEGFA expression, promoting the proliferation and metastasis of GC [54]. In our study, we identified that miR-4449 bound to circEIF4G3. Previous studies suggest that miR-4449 expression is upregulated in serum of patients with multiple myeloma and may serve as a potential biomarker [55]. Yan et al*.* suggest that miR-4449 promotes colorectal cancer cell proliferation via regulation of SOCS3/STAT3 signaling pathway [56]. However, the function and regulation of miR-4449 in GC remain largely unknown. We analyzed TCGA data and found that miR-4449 expression was elevated in GC tissues. Overexpression of miR-4449 promoted the proliferation, migration and invasion of GC cells, which implied that miR-4449 could function as an oncogene. Rescue experiments demonstrated that miR-4449 reserved the inhibitory effects of circEIF4G3 overexpression on GC cell proliferation, migration and invasion, indicating that circEIF4G3 may play a tumor suppressive role by sponging miR-4449. We further performed RNA-seq to identify the differentially expressed genes in circEIF4G3 overexpressing GC cells. Gene ontology analysis indicated that the altered genes by circEIF4G3 overexpression were enriched in multiple critical signaling pathways associated with cancer progression, including β-catenin signaling. By intersecting RNA-seq data and bioinformatic prediction results, we focused on SIK1, a protein of AMP-activated kinase (AMPK) family, which has been suggested as a tumor suppressor in many solid tumors, such as HCC [30], breast cancer [57], colorectal cancer [58]. Previous studies suggest that SIK1 disrupts the binding of β-catenin to the TBL1/TBLR1 complex, thereby inactivating the β-catenin signaling [30]. We confirmed that SIK1 overexpression suppressed GC progression while its knockdown reversed the tumor suppressive role of circEIF4G3 and identified a positive correlation between circEIF4G3 and SIK1 in tumor tissues of patients with GC, implying that SIK1 is an important downstream target of circEIF4G3. Moreover, we showed that circEIF4G3 increased SIK1 expression and decreased β-catenin expression and transactivity in GC cells, indicating that circEIF4G3 interfered with β-catenin signaling by modulating miR-4449/SIK1 axis.

## Conclusion

**Fig. 7:**
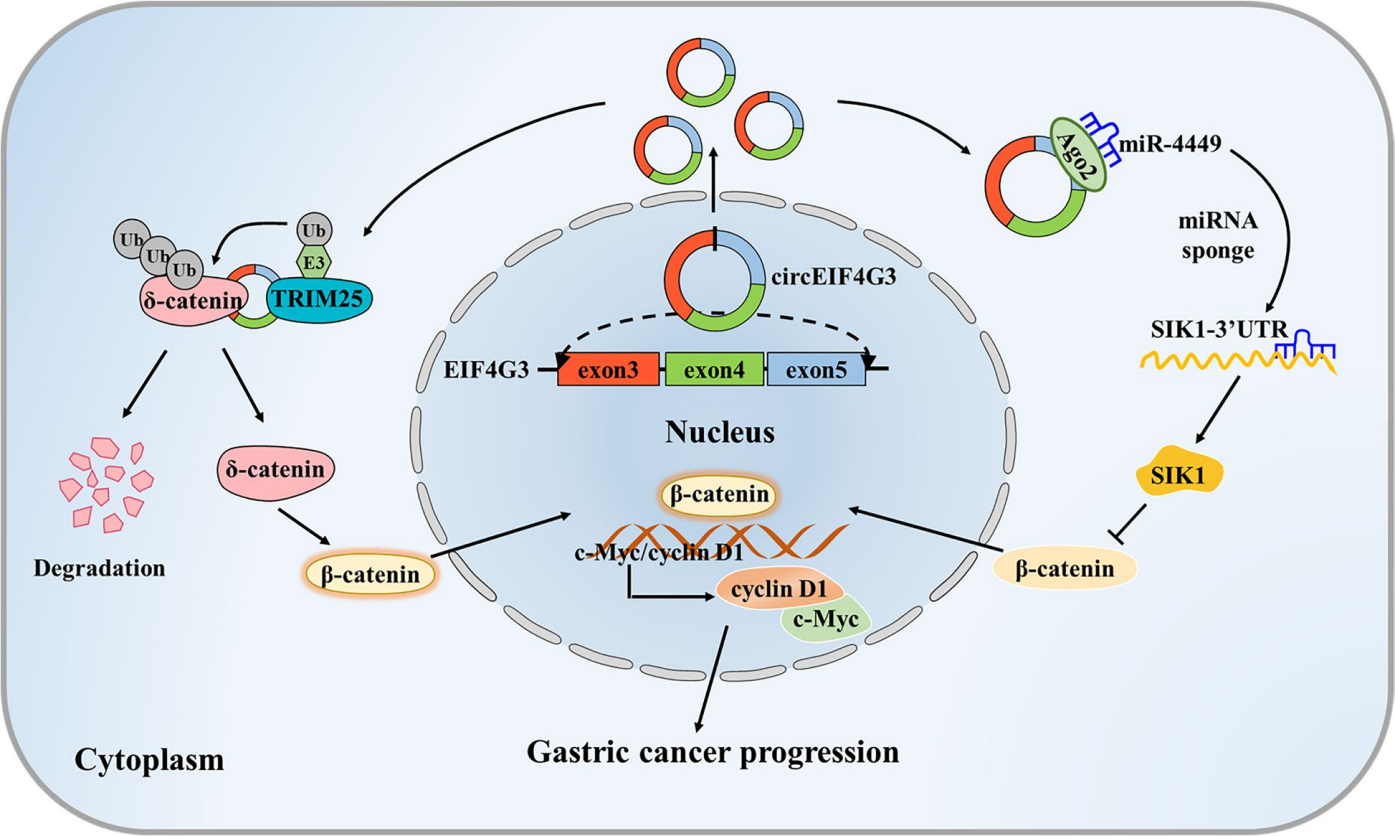
Proposed model for the roles and mechanisms of circEIF4G3 in GC progression. CircEIF4G3 destabilizes δ-catenin protein by enhancing TRIM25-mediated ubiquitin degradation and functions as a miRNA sponge to modulate miR-4449/SIK1 axis, which synergistically leads to the inactivation of β-catenin signaling and the inhibition of GC progression

Conclusively, our study revealed the role of a new circRNA, circEIF4G3, in GC progression and elucidated its mechanism of action (Fig. 7). CircEIF4G3 expression was downregulated in patients with GC and predicted poor prognosis. CircEIF4G3 destabilized δ-catenin by forming circEIF4G3/δ-catenin/TRIM25 RNA–protein ternary complexes, which consequently enhanced TRIM25-mediated ubiquitination and proteosomal degradation of δ-catenin. In addition, circEIF4G3 functioned as a sponge of miR-4449, and in turn promoted the expression of SIK1. The dual regulations subsequently led to the inactivation of β-catenin signaling and suppression of GC progression. Therefore, our findings provide a promising biomarker for GC prognosis and a potential target for GC therapy.

## Supplementary Information

**Additional file 1:** **Figure S1. **The expression and intracellular localization of circEIF4G3 in GC.(A) The common downregulated circRNAs in three GEO datasets were listed as indicated. (B) Nuclear/cytoplasm distribution of circEIF4G3 in GC cells.Actin and U6 were applied as positive controls. (C) qRT-PCR assays for the expression of circEIF4G3 in GC cell lines (HGC-27, AGS, BGC-823, SGC-7901, MGC-803, MKN-45,and NCI-N87) and a normal gastric mucosa epithelial cell line (GSE-1). (D) ROC curves for the diagnostic value of serum circEIF4G3 in GC. Data are shown as means±SD. ***P<0.001. **Figure S2. **CircEIF4G3 overexpression inhibits EMT in GC cells.(A) qRT-PCR was used to examine the efficiency of circEIF4G3 overexpression in GC cells. (B) Western blot and (C) qRT-PCR analyses of N-cadherin, E-cadherin, Vimentin, slug and cyclin D1 expression in control and circEIF4G3 overexpressing GC cells. **Figure S3. **CircEIF4G3 silencing promotes GC cell proliferation, migration and invasion in vitro.(A) Schematic illustration of specific circEIF4G3-targeting sites. (B) Efficiency of circEIF4G3 knockdown in GC cells by siRNAs was tested by qRT-PCR. (C) Cell counting assay,(D) Colony formation assay, and (E-F) Transwell migration and matrigel invasionassays for si-Scr and si-circEIF4G3 GC cells. (G) Western blot and (H) qRT-PCR assays to evaluate the expression of N-cadherin, E-cadherin, Vimentin and cyclin D1 mRNA and proteins in GC cells after circEIF4G3 knockdown. (I) Cell apoptosis assays for GC cells with or without circEIF4G3 knockdown. (J) Flow cytometry analyses of cell cycle distribution in si-Scr and si-circEIF4G3 GC cells. (K) Western blot analyses of β-catenin, c-Myc, and cyclin D1 expression in circEIF4G3 knockdown GC cells. Data are shown as means±SD (n = 3). *P<0.05, **P<0.01,***P<0.001; Scale bar=100 μm. **Figure S4. **δ-catenin overexpression promotes GC cell proliferation, migration, and invasion in vitro.(A) The protein level of δ-catenin overexpression in GC cells after transfection. (B) Cell counting assay, (C) Transwell migration, and (D) Matrigel invasion assays for GC cells with or without δ-catenin overexpression. Data are shown as means±SD. Scale bar=100 μm. **Figure S5. **δ-catenin partially rescues the inhibition of GC progression by circEIF4G3 overexpression.(A) Cell growth curve, (B) Colony formation,(C) Transwell migration, and (D) Matrigel invasion assays for circEIF4G3 overexpressing GC cells co-transfected with or without δ-catenin. Data are shown as means±SD. Scale bar=100 μm. (E) Western blot assays for protein levels of β-catenin and its downstream targets in circEIF4G3 overexpressing GC cells co-transfected with or without δ-catenin. (F) qRT-PCR analysis of circEIF4G3 expression and western blot assay for δ-catenin protein levels in paired tumor and non-tumor tissues. *P < 0.05,**P < 0.01. **Figure S6. **TRIM25 promotes ubiquitination and degradation of δ-catenin in a circEIF4G3-dependent manner. (A) Interaction of TRIM25 with circEIF4G3 was determined by TRAP assay and validated by western blot. (B) RNA FISH and immunofluorescence staining for the co-localization of circEIF4G3 (red) with TRIM25 (green) or δ-catenin (green) in GC cells. Scale bar=25 μm. (C) mRNA levels of δ-catenin in GC cells with TRIM25 overexpression. (D) The levels of ubiquitinated δ-catenin in TRIM25 overexpressing GC cells with circEIF4G3 knockdown. Data were expressed as means ± SD. **Figure S7. **SIK1 is a target of miR-4449. (A-B) The mRNA (A) and protein (B) levels of potential targets were detected in GC cells with circEIF4G3 (or miR-4449) overexpression and knockdown. (C) The correlation analysis between circEIF4G3 and HUNK or PINX.(D) The expression levels of β-catenin protein and downstream targets in GC cells with SIK1 overexpression were examined by western blot. (E) Relative luciferase activity of β-catenin was detected by dual-luciferase reporter assay**. ** (F-H) Cell counting (F), Transwellmigration (G), and Matrigel invasion (H) assays for GC cells with SIK1 overexpression. (I) The efficiency of SIK1 knockdown was determined by qRT-PCR.Scale bar =100 μm. **P< 0.01, ***P< 0.001. Data were expressed as means± SD. **Figure S8. **CircEIF4G3 overexpression inhibits δ-catenin while promotes SIK1 expression in vivo.The protein levels of δ-catenin and SIK1 in mouse tumor tissues were detected by western blot.

**Additional file 2:** **Supplementary Table 1. **Association between clinical features and circEIF4G3 expression of GC patients.** Supplementary Table 2. **Association between clinical features and circEIF4G3 expression of GC patients. **Supplementary Table 3.  **Primer sequences for qRT-PCR.** Supplementary Table 4. **Antibodies used in this study.

**Additional file 3:**

## Data Availability

All of the data and material in this paper are available when requested.

## Abbreviations

GC: Gastric cancer
circRNAs: Circular RNAs
RIP: RNA immunoprecipitation
RNA-FISH: RNA fluorescence in situ hybridization
TRAP: Tagged RNA affinity purification
TRIM25: Tripartite motif 25
CHX: Cycloheximide
CTNND1: Catenin Delta 1
Ub: Ubiquitin
