# Supplementary figures and images for: Circular RNA EIF4G3 suppresses gastric cancer progression through inhibition of β-catenin by promoting δ-catenin ubiquitin degradation and upregulating SIK1
Source: Mol Cancer. 2022 Jul 2;21:141. doi: 10.1186/s12943-022-01606-9 (PMC9250212; doi:10.1186/s12943-022-01606-9)

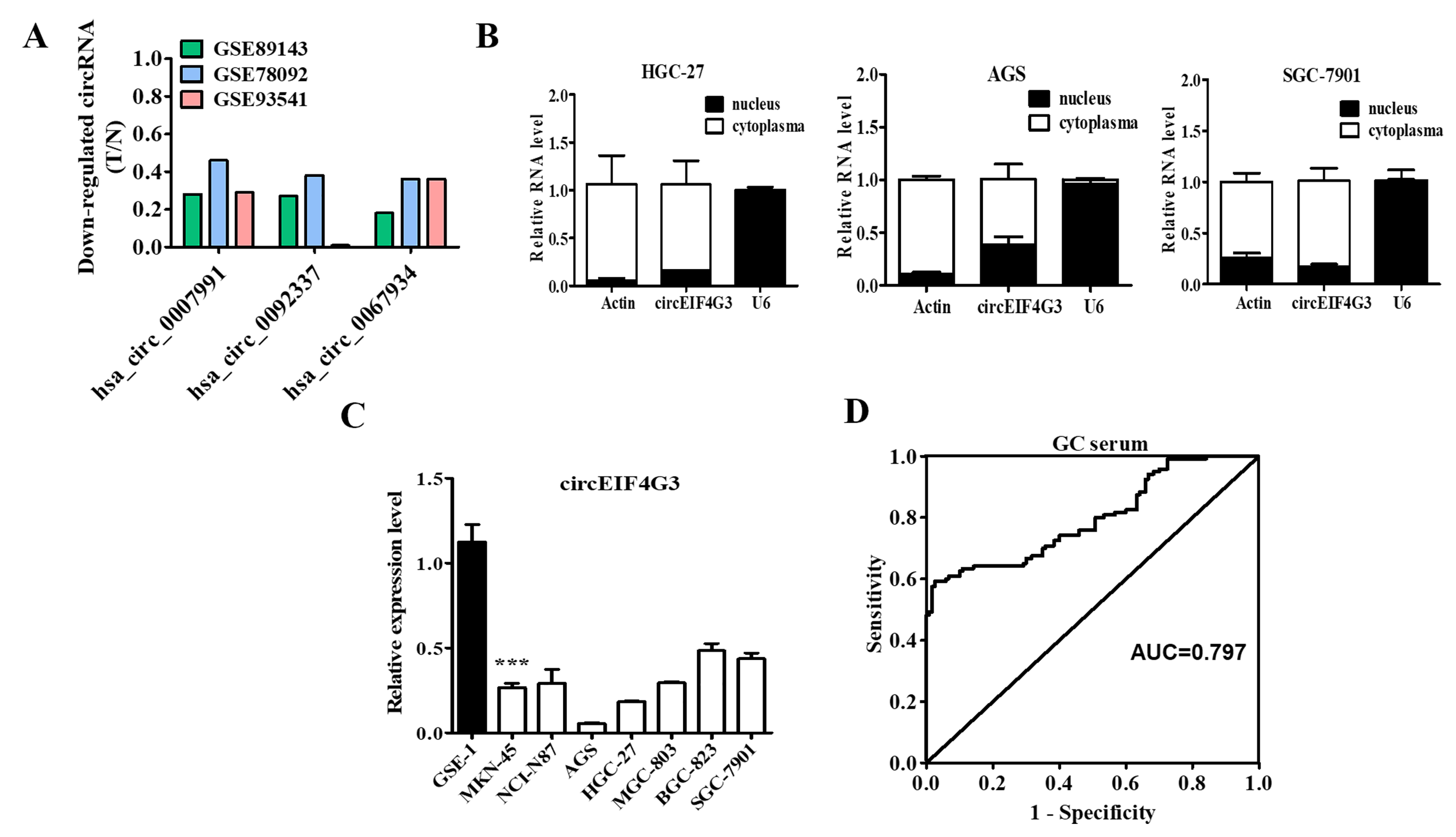

Supplement: Supplementary file 1 — Additional file 1: Figure S1. The expression and intracellular localization of circEIF4G3 in GC.(A) The common downregulated circRNAs in three GEO datasets were listed as indicated. (B) Nuclear/cytoplasm distribution of circEIF4G3 in GC cells.Actin and U6 were applied as positive controls. (C) qRT-PCR assays for the expression of circEIF4G3 in GC cell lines (HGC-27, AGS, BGC-823, SGC-7901, MGC-803, MKN-45,and NCI-N87) and a normal gastric mucosa epithelial cell line (GSE-1). (D) ROC curves for the diagnostic value of serum circEIF4G3 in GC. Data are shown as means±SD. ***P<0.001. Figure S2. CircEIF4G3 overexpression inhibits EMT in GC cells.(A) qRT-PCR was used to examine the efficiency of circEIF4G3 overexpression in GC cells. (B) Western blot and (C) qRT-PCR analyses of N-cadherin, E-cadherin, Vimentin, slug and cyclin D1 expression in control and circEIF4G3 overexpressing GC cells. Figure S3. CircEIF4G3 silencing promotes GC cell proliferation, migration and invasion in vitro.(A) Schematic illustration of specific circEIF4G3-targeting sites. (B) Efficiency of circEIF4G3 knockdown in GC cells by siRNAs was tested by qRT-PCR. (C) Cell counting assay,(D) Colony formation assay, and (E-F) Transwell migration and matrigel invasionassays for si-Scr and si-circEIF4G3 GC cells. (G) Western blot and (H) qRT-PCR assays to evaluate the expression of N-cadherin, E-cadherin, Vimentin and cyclin D1 mRNA and proteins in GC cells after circEIF4G3 knockdown. (I) Cell apoptosis assays for GC cells with or without circEIF4G3 knockdown. (J) Flow cytometry analyses of cell cycle distribution in si-Scr and si-circEIF4G3 GC cells. (K) Western blot analyses of β-catenin, c-Myc, and cyclin D1 expression in circEIF4G3 knockdown GC cells. Data are shown as means±SD (n = 3). *P<0.05, **P<0.01,***P<0.001; Scale bar=100 μm. Figure S4. δ-catenin overexpression promotes GC cell proliferation, migration, and invasion in vitro.(A) The protein level of δ-catenin overexpression in GC cells after tr [file 12943_2022_1606_MOESM1_ESM.zip › Fig.S1.tif]

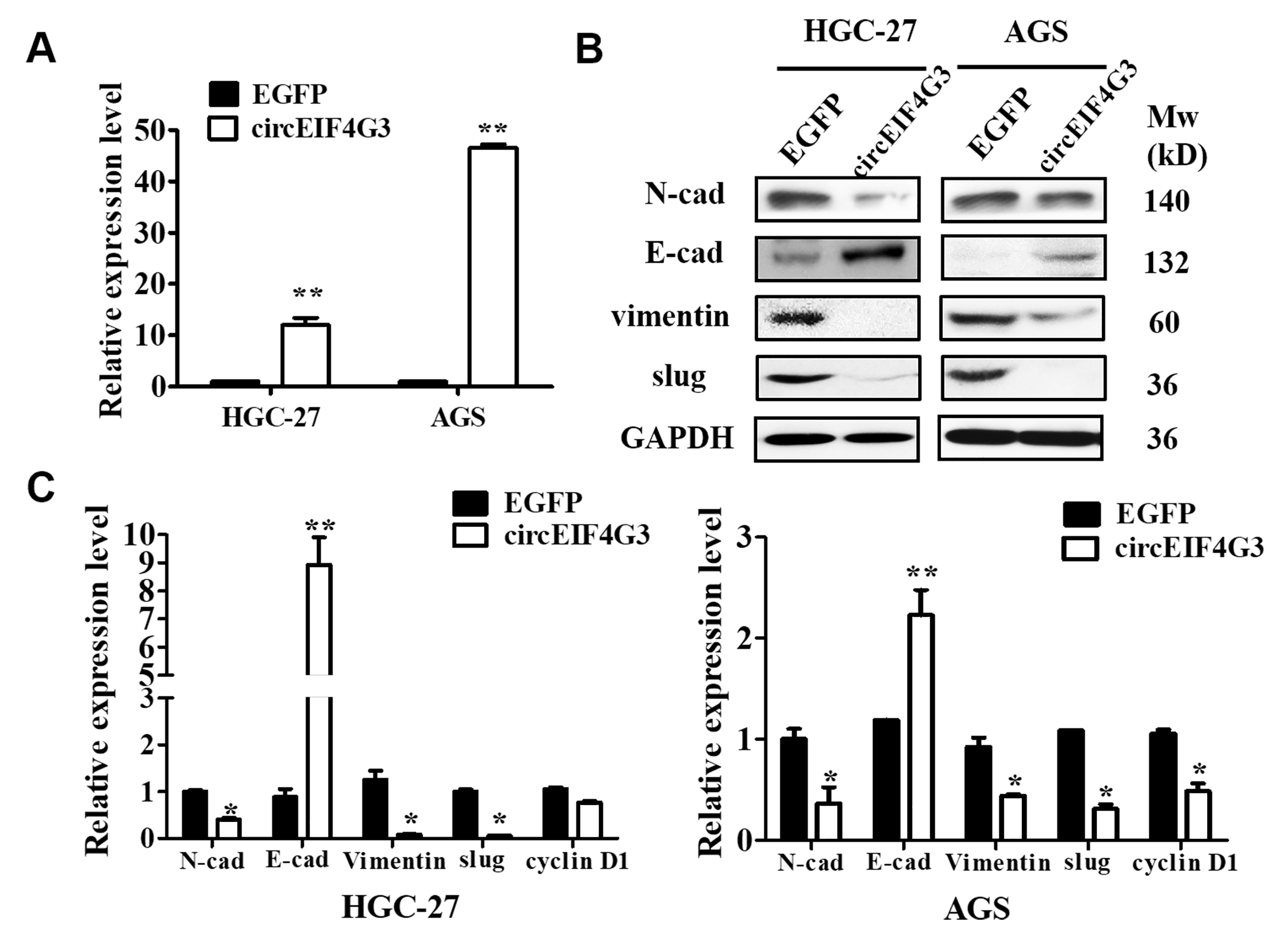

Supplement: Supplementary file 1 — Additional file 1: Figure S1. The expression and intracellular localization of circEIF4G3 in GC.(A) The common downregulated circRNAs in three GEO datasets were listed as indicated. (B) Nuclear/cytoplasm distribution of circEIF4G3 in GC cells.Actin and U6 were applied as positive controls. (C) qRT-PCR assays for the expression of circEIF4G3 in GC cell lines (HGC-27, AGS, BGC-823, SGC-7901, MGC-803, MKN-45,and NCI-N87) and a normal gastric mucosa epithelial cell line (GSE-1). (D) ROC curves for the diagnostic value of serum circEIF4G3 in GC. Data are shown as means±SD. ***P<0.001. Figure S2. CircEIF4G3 overexpression inhibits EMT in GC cells.(A) qRT-PCR was used to examine the efficiency of circEIF4G3 overexpression in GC cells. (B) Western blot and (C) qRT-PCR analyses of N-cadherin, E-cadherin, Vimentin, slug and cyclin D1 expression in control and circEIF4G3 overexpressing GC cells. Figure S3. CircEIF4G3 silencing promotes GC cell proliferation, migration and invasion in vitro.(A) Schematic illustration of specific circEIF4G3-targeting sites. (B) Efficiency of circEIF4G3 knockdown in GC cells by siRNAs was tested by qRT-PCR. (C) Cell counting assay,(D) Colony formation assay, and (E-F) Transwell migration and matrigel invasionassays for si-Scr and si-circEIF4G3 GC cells. (G) Western blot and (H) qRT-PCR assays to evaluate the expression of N-cadherin, E-cadherin, Vimentin and cyclin D1 mRNA and proteins in GC cells after circEIF4G3 knockdown. (I) Cell apoptosis assays for GC cells with or without circEIF4G3 knockdown. (J) Flow cytometry analyses of cell cycle distribution in si-Scr and si-circEIF4G3 GC cells. (K) Western blot analyses of β-catenin, c-Myc, and cyclin D1 expression in circEIF4G3 knockdown GC cells. Data are shown as means±SD (n = 3). *P<0.05, **P<0.01,***P<0.001; Scale bar=100 μm. Figure S4. δ-catenin overexpression promotes GC cell proliferation, migration, and invasion in vitro.(A) The protein level of δ-catenin overexpression in GC cells after tr [file 12943_2022_1606_MOESM1_ESM.zip › Fig.S2.tif]

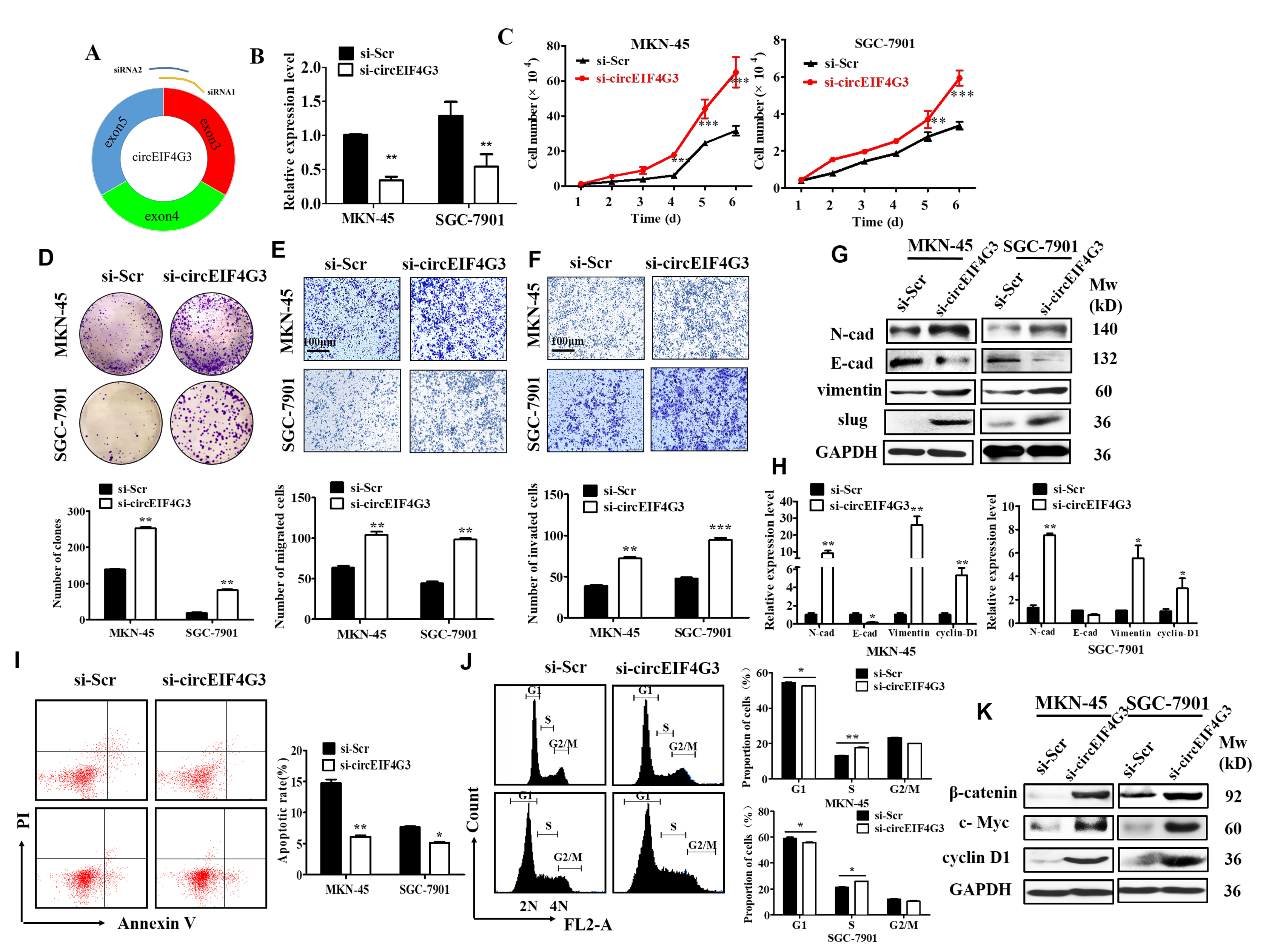

Supplement: Supplementary file 1 — Additional file 1: Figure S1. The expression and intracellular localization of circEIF4G3 in GC.(A) The common downregulated circRNAs in three GEO datasets were listed as indicated. (B) Nuclear/cytoplasm distribution of circEIF4G3 in GC cells.Actin and U6 were applied as positive controls. (C) qRT-PCR assays for the expression of circEIF4G3 in GC cell lines (HGC-27, AGS, BGC-823, SGC-7901, MGC-803, MKN-45,and NCI-N87) and a normal gastric mucosa epithelial cell line (GSE-1). (D) ROC curves for the diagnostic value of serum circEIF4G3 in GC. Data are shown as means±SD. ***P<0.001. Figure S2. CircEIF4G3 overexpression inhibits EMT in GC cells.(A) qRT-PCR was used to examine the efficiency of circEIF4G3 overexpression in GC cells. (B) Western blot and (C) qRT-PCR analyses of N-cadherin, E-cadherin, Vimentin, slug and cyclin D1 expression in control and circEIF4G3 overexpressing GC cells. Figure S3. CircEIF4G3 silencing promotes GC cell proliferation, migration and invasion in vitro.(A) Schematic illustration of specific circEIF4G3-targeting sites. (B) Efficiency of circEIF4G3 knockdown in GC cells by siRNAs was tested by qRT-PCR. (C) Cell counting assay,(D) Colony formation assay, and (E-F) Transwell migration and matrigel invasionassays for si-Scr and si-circEIF4G3 GC cells. (G) Western blot and (H) qRT-PCR assays to evaluate the expression of N-cadherin, E-cadherin, Vimentin and cyclin D1 mRNA and proteins in GC cells after circEIF4G3 knockdown. (I) Cell apoptosis assays for GC cells with or without circEIF4G3 knockdown. (J) Flow cytometry analyses of cell cycle distribution in si-Scr and si-circEIF4G3 GC cells. (K) Western blot analyses of β-catenin, c-Myc, and cyclin D1 expression in circEIF4G3 knockdown GC cells. Data are shown as means±SD (n = 3). *P<0.05, **P<0.01,***P<0.001; Scale bar=100 μm. Figure S4. δ-catenin overexpression promotes GC cell proliferation, migration, and invasion in vitro.(A) The protein level of δ-catenin overexpression in GC cells after tr [file 12943_2022_1606_MOESM1_ESM.zip › Fig.S3.tif]

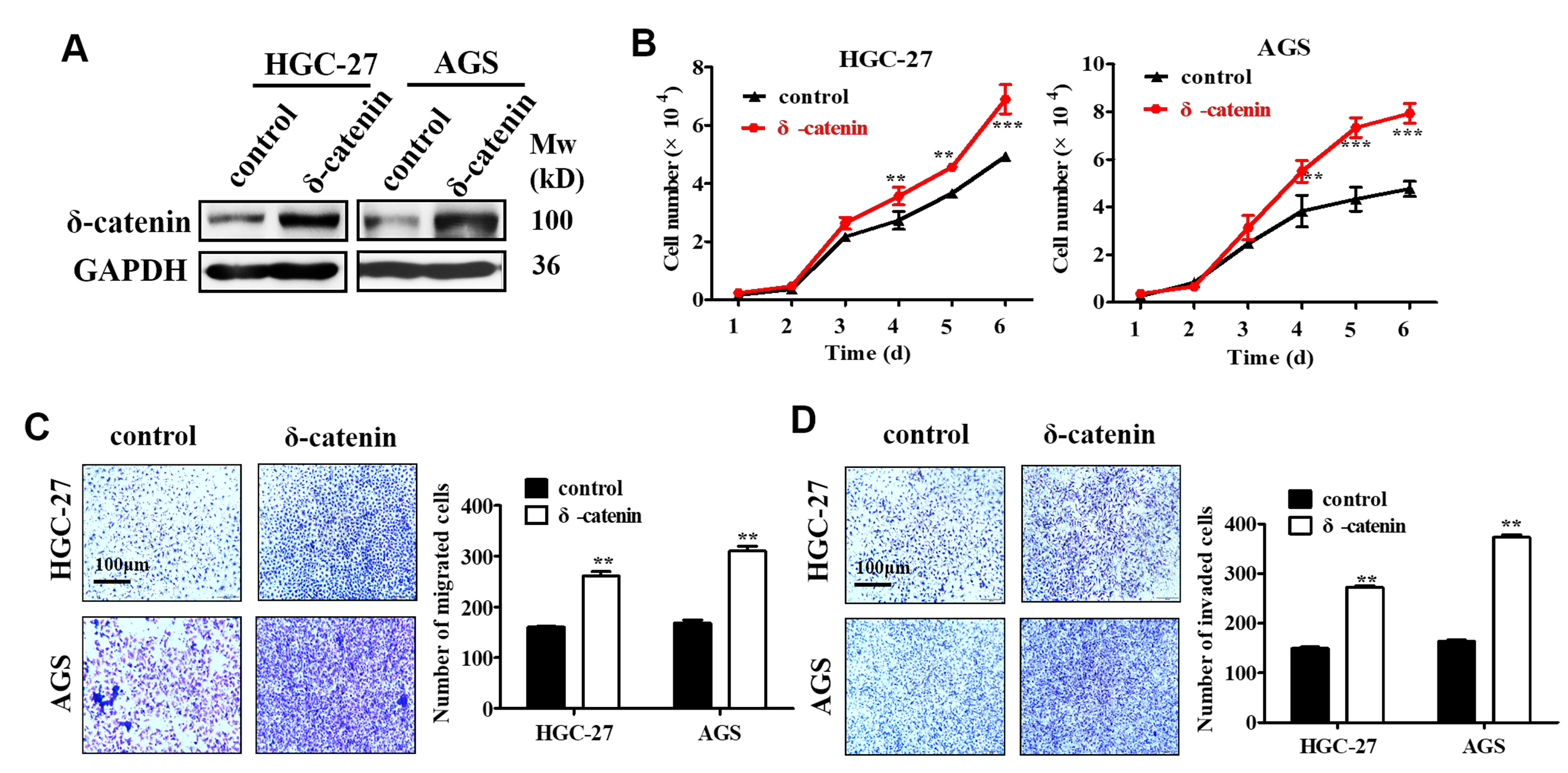

Supplement: Supplementary file 1 — Additional file 1: Figure S1. The expression and intracellular localization of circEIF4G3 in GC.(A) The common downregulated circRNAs in three GEO datasets were listed as indicated. (B) Nuclear/cytoplasm distribution of circEIF4G3 in GC cells.Actin and U6 were applied as positive controls. (C) qRT-PCR assays for the expression of circEIF4G3 in GC cell lines (HGC-27, AGS, BGC-823, SGC-7901, MGC-803, MKN-45,and NCI-N87) and a normal gastric mucosa epithelial cell line (GSE-1). (D) ROC curves for the diagnostic value of serum circEIF4G3 in GC. Data are shown as means±SD. ***P<0.001. Figure S2. CircEIF4G3 overexpression inhibits EMT in GC cells.(A) qRT-PCR was used to examine the efficiency of circEIF4G3 overexpression in GC cells. (B) Western blot and (C) qRT-PCR analyses of N-cadherin, E-cadherin, Vimentin, slug and cyclin D1 expression in control and circEIF4G3 overexpressing GC cells. Figure S3. CircEIF4G3 silencing promotes GC cell proliferation, migration and invasion in vitro.(A) Schematic illustration of specific circEIF4G3-targeting sites. (B) Efficiency of circEIF4G3 knockdown in GC cells by siRNAs was tested by qRT-PCR. (C) Cell counting assay,(D) Colony formation assay, and (E-F) Transwell migration and matrigel invasionassays for si-Scr and si-circEIF4G3 GC cells. (G) Western blot and (H) qRT-PCR assays to evaluate the expression of N-cadherin, E-cadherin, Vimentin and cyclin D1 mRNA and proteins in GC cells after circEIF4G3 knockdown. (I) Cell apoptosis assays for GC cells with or without circEIF4G3 knockdown. (J) Flow cytometry analyses of cell cycle distribution in si-Scr and si-circEIF4G3 GC cells. (K) Western blot analyses of β-catenin, c-Myc, and cyclin D1 expression in circEIF4G3 knockdown GC cells. Data are shown as means±SD (n = 3). *P<0.05, **P<0.01,***P<0.001; Scale bar=100 μm. Figure S4. δ-catenin overexpression promotes GC cell proliferation, migration, and invasion in vitro.(A) The protein level of δ-catenin overexpression in GC cells after tr [file 12943_2022_1606_MOESM1_ESM.zip › Fig.S4.tif]

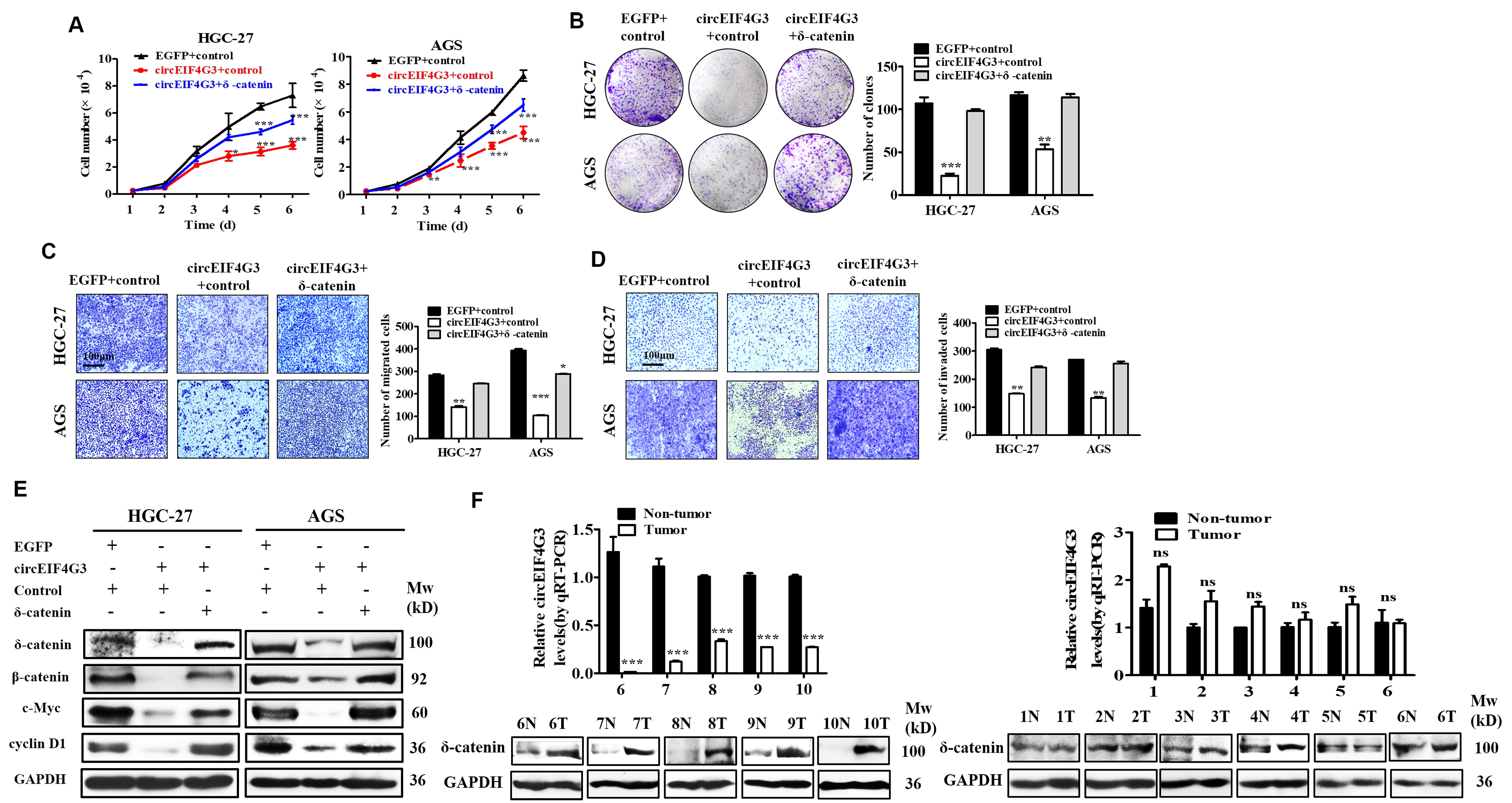

Supplement: Supplementary file 1 — Additional file 1: Figure S1. The expression and intracellular localization of circEIF4G3 in GC.(A) The common downregulated circRNAs in three GEO datasets were listed as indicated. (B) Nuclear/cytoplasm distribution of circEIF4G3 in GC cells.Actin and U6 were applied as positive controls. (C) qRT-PCR assays for the expression of circEIF4G3 in GC cell lines (HGC-27, AGS, BGC-823, SGC-7901, MGC-803, MKN-45,and NCI-N87) and a normal gastric mucosa epithelial cell line (GSE-1). (D) ROC curves for the diagnostic value of serum circEIF4G3 in GC. Data are shown as means±SD. ***P<0.001. Figure S2. CircEIF4G3 overexpression inhibits EMT in GC cells.(A) qRT-PCR was used to examine the efficiency of circEIF4G3 overexpression in GC cells. (B) Western blot and (C) qRT-PCR analyses of N-cadherin, E-cadherin, Vimentin, slug and cyclin D1 expression in control and circEIF4G3 overexpressing GC cells. Figure S3. CircEIF4G3 silencing promotes GC cell proliferation, migration and invasion in vitro.(A) Schematic illustration of specific circEIF4G3-targeting sites. (B) Efficiency of circEIF4G3 knockdown in GC cells by siRNAs was tested by qRT-PCR. (C) Cell counting assay,(D) Colony formation assay, and (E-F) Transwell migration and matrigel invasionassays for si-Scr and si-circEIF4G3 GC cells. (G) Western blot and (H) qRT-PCR assays to evaluate the expression of N-cadherin, E-cadherin, Vimentin and cyclin D1 mRNA and proteins in GC cells after circEIF4G3 knockdown. (I) Cell apoptosis assays for GC cells with or without circEIF4G3 knockdown. (J) Flow cytometry analyses of cell cycle distribution in si-Scr and si-circEIF4G3 GC cells. (K) Western blot analyses of β-catenin, c-Myc, and cyclin D1 expression in circEIF4G3 knockdown GC cells. Data are shown as means±SD (n = 3). *P<0.05, **P<0.01,***P<0.001; Scale bar=100 μm. Figure S4. δ-catenin overexpression promotes GC cell proliferation, migration, and invasion in vitro.(A) The protein level of δ-catenin overexpression in GC cells after tr [file 12943_2022_1606_MOESM1_ESM.zip › Fig.S5.tif]

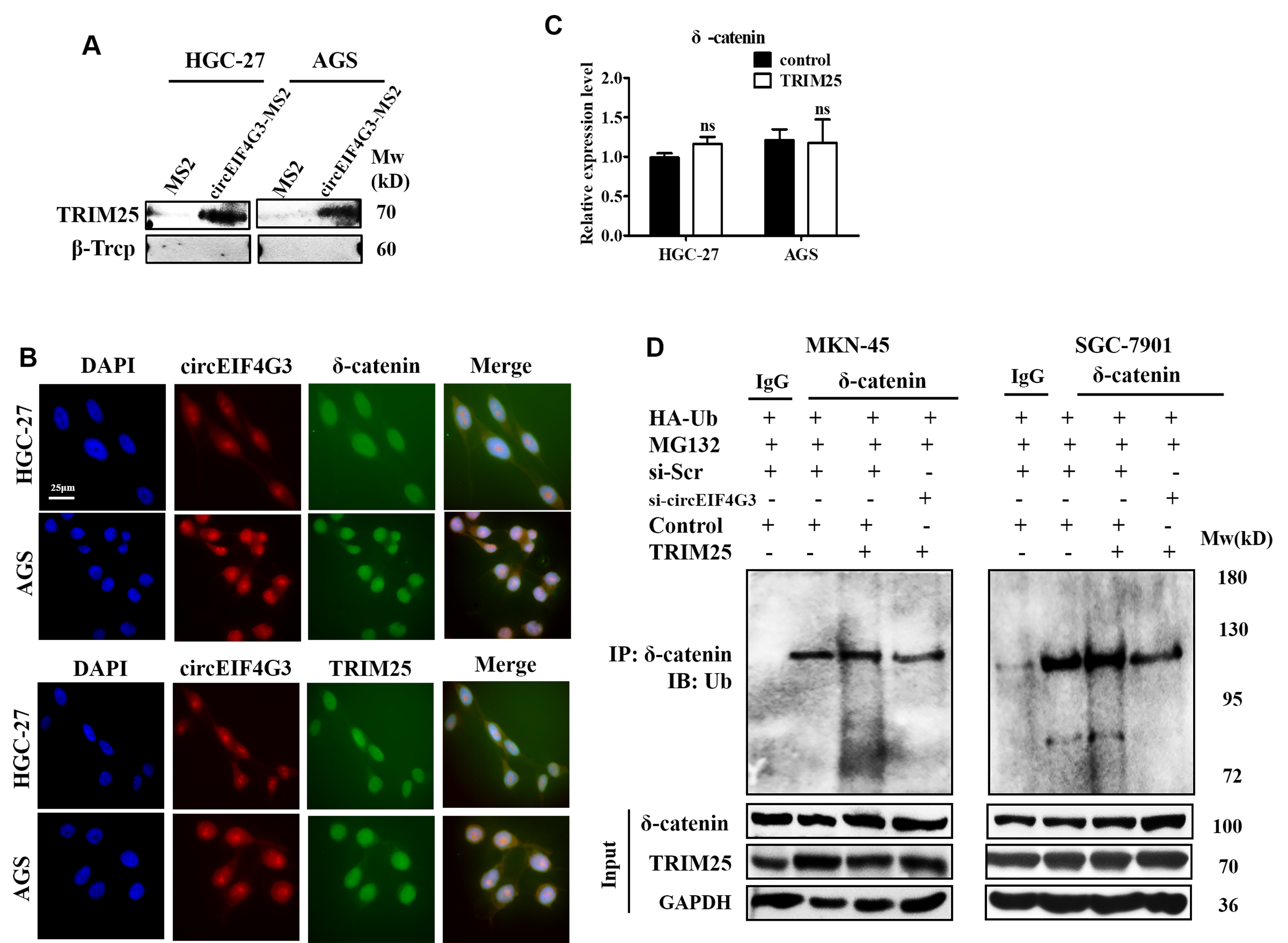

Supplement: Supplementary file 1 — Additional file 1: Figure S1. The expression and intracellular localization of circEIF4G3 in GC.(A) The common downregulated circRNAs in three GEO datasets were listed as indicated. (B) Nuclear/cytoplasm distribution of circEIF4G3 in GC cells.Actin and U6 were applied as positive controls. (C) qRT-PCR assays for the expression of circEIF4G3 in GC cell lines (HGC-27, AGS, BGC-823, SGC-7901, MGC-803, MKN-45,and NCI-N87) and a normal gastric mucosa epithelial cell line (GSE-1). (D) ROC curves for the diagnostic value of serum circEIF4G3 in GC. Data are shown as means±SD. ***P<0.001. Figure S2. CircEIF4G3 overexpression inhibits EMT in GC cells.(A) qRT-PCR was used to examine the efficiency of circEIF4G3 overexpression in GC cells. (B) Western blot and (C) qRT-PCR analyses of N-cadherin, E-cadherin, Vimentin, slug and cyclin D1 expression in control and circEIF4G3 overexpressing GC cells. Figure S3. CircEIF4G3 silencing promotes GC cell proliferation, migration and invasion in vitro.(A) Schematic illustration of specific circEIF4G3-targeting sites. (B) Efficiency of circEIF4G3 knockdown in GC cells by siRNAs was tested by qRT-PCR. (C) Cell counting assay,(D) Colony formation assay, and (E-F) Transwell migration and matrigel invasionassays for si-Scr and si-circEIF4G3 GC cells. (G) Western blot and (H) qRT-PCR assays to evaluate the expression of N-cadherin, E-cadherin, Vimentin and cyclin D1 mRNA and proteins in GC cells after circEIF4G3 knockdown. (I) Cell apoptosis assays for GC cells with or without circEIF4G3 knockdown. (J) Flow cytometry analyses of cell cycle distribution in si-Scr and si-circEIF4G3 GC cells. (K) Western blot analyses of β-catenin, c-Myc, and cyclin D1 expression in circEIF4G3 knockdown GC cells. Data are shown as means±SD (n = 3). *P<0.05, **P<0.01,***P<0.001; Scale bar=100 μm. Figure S4. δ-catenin overexpression promotes GC cell proliferation, migration, and invasion in vitro.(A) The protein level of δ-catenin overexpression in GC cells after tr [file 12943_2022_1606_MOESM1_ESM.zip › Fig.S6.tif]

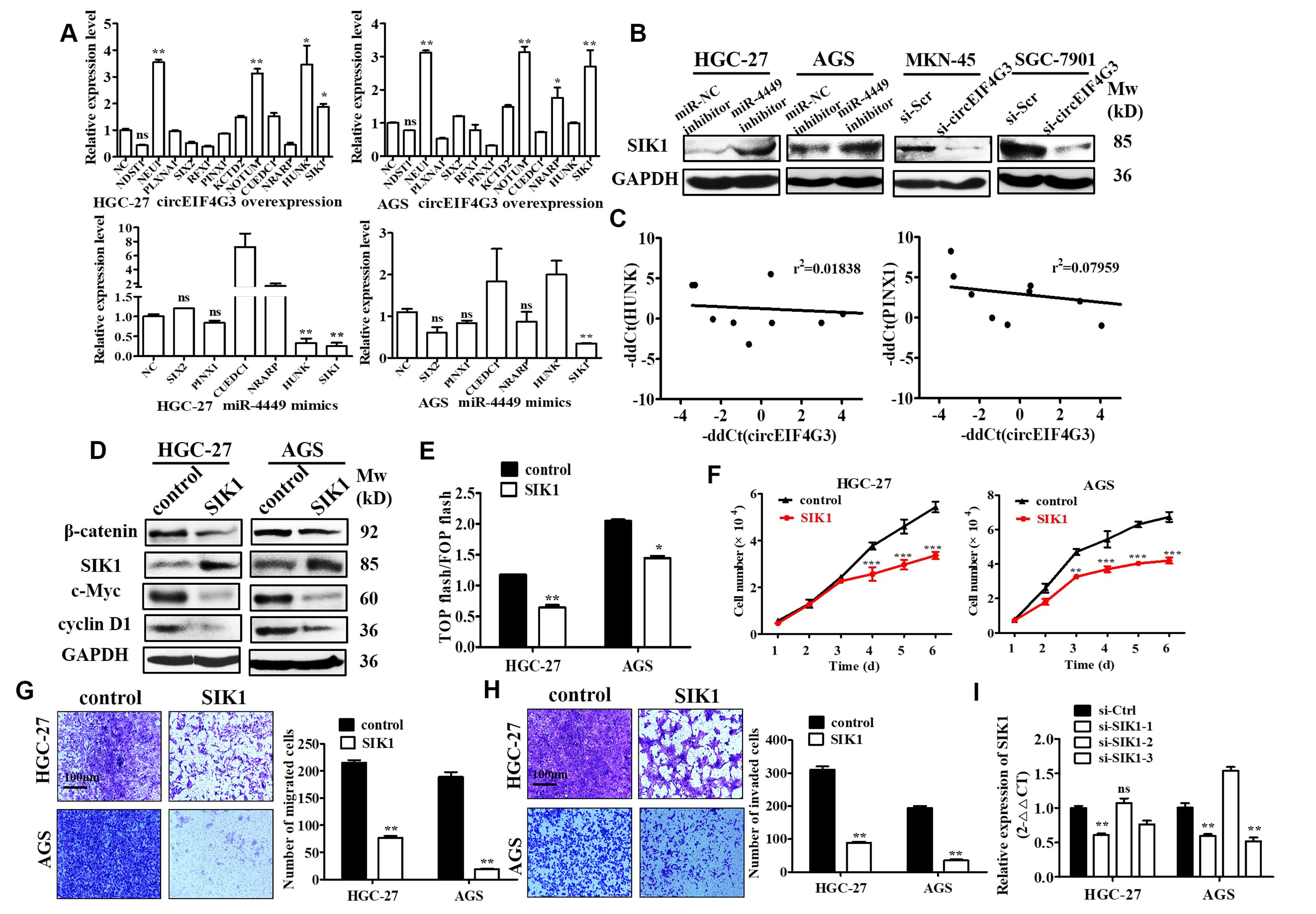

Supplement: Supplementary file 1 — Additional file 1: Figure S1. The expression and intracellular localization of circEIF4G3 in GC.(A) The common downregulated circRNAs in three GEO datasets were listed as indicated. (B) Nuclear/cytoplasm distribution of circEIF4G3 in GC cells.Actin and U6 were applied as positive controls. (C) qRT-PCR assays for the expression of circEIF4G3 in GC cell lines (HGC-27, AGS, BGC-823, SGC-7901, MGC-803, MKN-45,and NCI-N87) and a normal gastric mucosa epithelial cell line (GSE-1). (D) ROC curves for the diagnostic value of serum circEIF4G3 in GC. Data are shown as means±SD. ***P<0.001. Figure S2. CircEIF4G3 overexpression inhibits EMT in GC cells.(A) qRT-PCR was used to examine the efficiency of circEIF4G3 overexpression in GC cells. (B) Western blot and (C) qRT-PCR analyses of N-cadherin, E-cadherin, Vimentin, slug and cyclin D1 expression in control and circEIF4G3 overexpressing GC cells. Figure S3. CircEIF4G3 silencing promotes GC cell proliferation, migration and invasion in vitro.(A) Schematic illustration of specific circEIF4G3-targeting sites. (B) Efficiency of circEIF4G3 knockdown in GC cells by siRNAs was tested by qRT-PCR. (C) Cell counting assay,(D) Colony formation assay, and (E-F) Transwell migration and matrigel invasionassays for si-Scr and si-circEIF4G3 GC cells. (G) Western blot and (H) qRT-PCR assays to evaluate the expression of N-cadherin, E-cadherin, Vimentin and cyclin D1 mRNA and proteins in GC cells after circEIF4G3 knockdown. (I) Cell apoptosis assays for GC cells with or without circEIF4G3 knockdown. (J) Flow cytometry analyses of cell cycle distribution in si-Scr and si-circEIF4G3 GC cells. (K) Western blot analyses of β-catenin, c-Myc, and cyclin D1 expression in circEIF4G3 knockdown GC cells. Data are shown as means±SD (n = 3). *P<0.05, **P<0.01,***P<0.001; Scale bar=100 μm. Figure S4. δ-catenin overexpression promotes GC cell proliferation, migration, and invasion in vitro.(A) The protein level of δ-catenin overexpression in GC cells after tr [file 12943_2022_1606_MOESM1_ESM.zip › Fig.S7.tif]

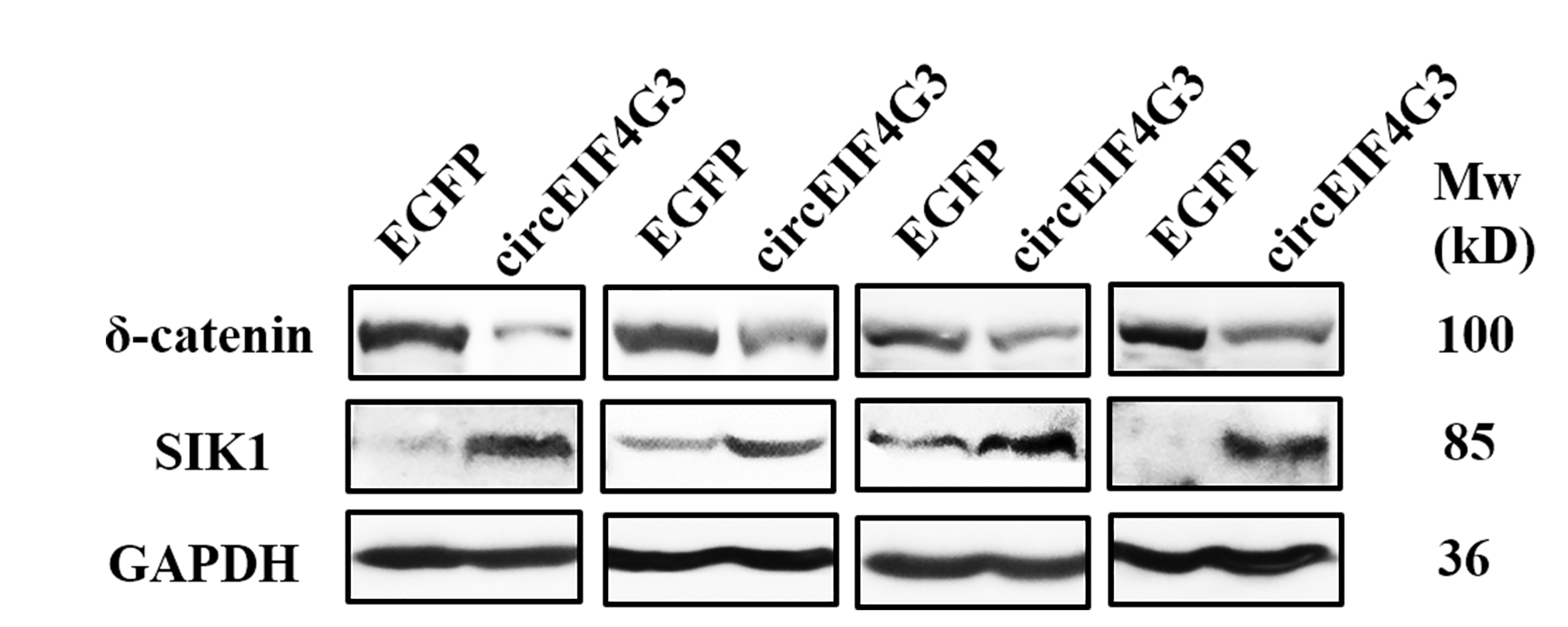

Supplement: Supplementary file 1 — Additional file 1: Figure S1. The expression and intracellular localization of circEIF4G3 in GC.(A) The common downregulated circRNAs in three GEO datasets were listed as indicated. (B) Nuclear/cytoplasm distribution of circEIF4G3 in GC cells.Actin and U6 were applied as positive controls. (C) qRT-PCR assays for the expression of circEIF4G3 in GC cell lines (HGC-27, AGS, BGC-823, SGC-7901, MGC-803, MKN-45,and NCI-N87) and a normal gastric mucosa epithelial cell line (GSE-1). (D) ROC curves for the diagnostic value of serum circEIF4G3 in GC. Data are shown as means±SD. ***P<0.001. Figure S2. CircEIF4G3 overexpression inhibits EMT in GC cells.(A) qRT-PCR was used to examine the efficiency of circEIF4G3 overexpression in GC cells. (B) Western blot and (C) qRT-PCR analyses of N-cadherin, E-cadherin, Vimentin, slug and cyclin D1 expression in control and circEIF4G3 overexpressing GC cells. Figure S3. CircEIF4G3 silencing promotes GC cell proliferation, migration and invasion in vitro.(A) Schematic illustration of specific circEIF4G3-targeting sites. (B) Efficiency of circEIF4G3 knockdown in GC cells by siRNAs was tested by qRT-PCR. (C) Cell counting assay,(D) Colony formation assay, and (E-F) Transwell migration and matrigel invasionassays for si-Scr and si-circEIF4G3 GC cells. (G) Western blot and (H) qRT-PCR assays to evaluate the expression of N-cadherin, E-cadherin, Vimentin and cyclin D1 mRNA and proteins in GC cells after circEIF4G3 knockdown. (I) Cell apoptosis assays for GC cells with or without circEIF4G3 knockdown. (J) Flow cytometry analyses of cell cycle distribution in si-Scr and si-circEIF4G3 GC cells. (K) Western blot analyses of β-catenin, c-Myc, and cyclin D1 expression in circEIF4G3 knockdown GC cells. Data are shown as means±SD (n = 3). *P<0.05, **P<0.01,***P<0.001; Scale bar=100 μm. Figure S4. δ-catenin overexpression promotes GC cell proliferation, migration, and invasion in vitro.(A) The protein level of δ-catenin overexpression in GC cells after tr [file 12943_2022_1606_MOESM1_ESM.zip › Fig.S8.tif]
