# Supplementary material for: Circular RNA EIF4G3 suppresses gastric cancer progression through inhibition of β-catenin by promoting δ-catenin ubiquitin degradation and upregulating SIK1
Source: Mol Cancer. 2022 Jul 2;21:141. doi: 10.1186/s12943-022-01606-9 (PMC9250212; doi:10.1186/s12943-022-01606-9)
Supplement: Supplementary file 2 — Additional file 2: Supplementary Table 1. Association between clinical features and circEIF4G3 expression of GC patients. Supplementary Table 2. Association between clinical features and circEIF4G3 expression of GC patients. Supplementary Table 3. Primer sequences for qRT-PCR. Supplementary Table 4. Antibodies used in this study. [file 12943_2022_1606_MOESM2_ESM.docx]

**Supplementary Materials and Methods**

**RNA extraction and quantitative real-time PCR (qRT-PCR)**

The total RNA was extracted from tissues or cultured cells with Trizol following the manufacture’s protocol (Invitrogen, MA, USA). Total RNA in serum was separated by utilizing miRNeasy serum/plasma kit according to the manufacturer’s instructions (Qiagen, CA, USA). Then HiScript 1st Strand cDNA Synthesis Kit (Vazyme) was used to reversely transcribe the RNA into cDNA. QRT-PCR reactions were performed using the SYBR Green PCR Master Mix (Vazyme, Nanjing, China). The results were normalized to GAPDH and expression fold change was calculated according to the 2^−ΔΔCt^ method. The primers used for real-time PCR are shown in Additional Table 3.

**Western blot**

Total proteins were extracted using RIPA lysis buffer supplemented with protease inhibitors (Pierce), separated on 12% SDS-polyacrylamide gels, and transferred into polyvinylidene fluoride (PVDF) membranes (Millipore, Billerica, MA, USA). After blocking with 2% non-fat milk, the above membranes were incubated with corresponding primary antibodies at 4℃ overnight. Following extensive washing with TBST, the membranes were incubated with secondary antibodies for 2 h at room temperature. Protein bands were visualized by chemiluminescence detection kit (Vazyme). The information of antibodies used is shown in Additional Table 4.

**RNase R resistance assay**

For RNase R treatment, 1μg of total RNA was incubated at 37℃ with or without 2.5U of RNase R (Epicentre Biotechnologies, Madison, WI). Reverse transcription (RT) was performed by random hexamers and SuperScript (Vazyme) and quantitative PCR (qPCR) was performed by SYBR Green master mix (Vazyme).

**RNA fluorescence in situ hybridization (FISH)**

The subcellular distribution of circRNA was performed by Fluorescent In Situ Hybridization Kit (GenePharma, Shanghai, China) according to the manufacturer’s guidelines. Cy-3-conjugated circEIF4G3 probes were incubated with cells at 37℃ in the dark overnight. Images were captured using confocal microscopy. The sequence of the detection probe was as follows: 5’-Cy- GCACAGGTCCTCTAGGAATTCGGAGAACGGGTTTGAGGTT-3’.

**Cell counting assay and cell colony formation assay**

At 24 h after transfection, 1×10^4^/cells were seeded in 24-well plates and counted for 6 days. For the colony formation assay, 1×10^3^ transfected cells were seeded in 6-well plates and cultured for 14 days. The cell colonies were fixed with 4% paraformaldehyde for 15 min and stained with 0.1% crystal violet at room temperature. Cell colonies were counted and imaged. The assay was repeated three times in duplicate.

**Cell cycle analysis**

Cell cycle analysis was performed with a cell cycle detection kit (Fcmacs, Nanjing, China). The transfected cells were collected and fixed in 95% ethanol overnight. Then, the cells were stained with 50 μg/ml propidium iodide (PI) for 30 min in dark. Finally, the cell cycle distribution was analyzed with a flow cytometer (BD, FACS Calibur) via CellQuest software.

**Cell apoptosis assay**

Cell apoptosis assay was performed by Annexin V-Alexa Fluor 647/PI apoptosis detection kit (Fcmacs, Nanjing, China). The transfected cells were collected and re-suspended in binding buffer. Subsequently, the cells were stained with Annexin V-Alexa Fluor 647 and PI and then analyzed by flow cytometry (FACScan; BD Biosciences) using CellQuest software (BD Biosciences, San Jose, CA, USA).

**Transwell migration and matrigel invasion assays**

Transwell assay was carried out to measure cell migration and invasion using the 24-well transwell chambers (8μm pore; Corning, MA, USA) with or without Matrigel (BD Biosciences, NY, USA). The transfected cells were seeded into the upper chamber in 200 μL serum-free medium, while the lower chamber was placed with 10% FBS medium as a chemoattractant. The cells were incubated at 37℃ in 5% CO_2_ for 24 h for the migration assay or 48 h for the invasion assay. Then, the cells were fixed with 4% paraformaldehyde, stained with crystal violet solution, and photographed under a microscope at 100× magnification. The experiments were repeated for at least three times.
