# Supplementary material for: Circular RNA EIF4G3 suppresses gastric cancer progression through inhibition of β-catenin by promoting δ-catenin ubiquitin degradation and upregulating SIK1
Source: Mol Cancer. 2022 Jul 2;21:141. doi: 10.1186/s12943-022-01606-9 (PMC9250212; doi:10.1186/s12943-022-01606-9)
Supplement: Supplementary file 3 — Additional file 3: [file 12943_2022_1606_MOESM3_ESM.zip › 2-Supplementary Table 1.docx]

**Supplementary Table 2: Association between clinical features and circEIF4G3 expression of GC patients**

| **Features** | **Number** | **circEIF4G3 expression** | | **P value** |
| --- | --- | --- | --- | --- |
|  |  | **High** | **Low** |  |
| **Gender** |  |  |  | 0.675 |
| Male | 66 | 17 | 49 |  |
| Female | 32 | 7 | 25 |  |
| **Age, years** |  |  |  | 0.232 |
| ＜60 | 30 | 5 | 25 |  |
| ≥60 | 68 | 19 | 49 |  |
| **Tumor size (cm)** |  |  |  | 0.631 |
| ＜5 | 53 | 14 | 39 |  |
| ≥5 | 45 | 10 | 35 |  |
| **Venous invasion*** |  |  |  | 0.047 |
| Absent | 74 | 19 | 55 |  |
| Present | 17 | 1 | 16 |  |
| **Invasion depth*** |  |  |  | 0.371 |
| T1 and T2 | 8 | 3 | 5 |  |
| T3 and T4 | 88 | 20 | 68 |  |
| **Tumor location** |  | |  | 0.704 |
| Antrum | 27 | 6 | 21 |  |
| Body  Angulus  Cardia  Others | 12  9  47  3 | 3  2  12  1 | 9  7  35  2 |  |
| **TNM stage*** |  |  |  | 0.048 |
| I and II | 34 | 12 | 22 |  |
| III and IV | 59 | 10 | 49 |  |

*** missing case**

*** missing case**
