# Supplementary material for: Circular RNA EIF4G3 suppresses gastric cancer progression through inhibition of β-catenin by promoting δ-catenin ubiquitin degradation and upregulating SIK1
Source: Mol Cancer. 2022 Jul 2;21:141. doi: 10.1186/s12943-022-01606-9 (PMC9250212; doi:10.1186/s12943-022-01606-9)
Supplement: Supplementary file 3 — Additional file 3: [file 12943_2022_1606_MOESM3_ESM.zip › 2-Supplementary Table 2.docx]

**Supplementary Table 2: Association between circEIF4G3 expression and clinicopathological features of GC serum**

| **Features** | **Number** | **CircEIF4G3 expression** | | **P value** |
| --- | --- | --- | --- | --- |
|  |  | **High** | **Low** |  |
| **Gender*** |  |  |  | 0.365 |
| Male | 67 | 20 | 47 |  |
| Female | 37 | 8 | 29 |  |
| **Age, years*** |  |  |  | 0.478 |
| ＜60 | 21 | 7 | 14 |  |
| ≥60 | 82 | 21 | 61 |  |
| **Tumor size (cm)*** |  |  |  | 0.968 |
| ＜5 | 63 | 18 | 45 |  |
| ≥5 | 38 | 11 | 27 |  |
| **Lymphatic metastasis** |  |  |  | 0.043 |
| N0 | 20 | 17 | 3 |  |
| N1-3 | 88 | 33 | 55 |  |
| **Distant metastasis** |  |  |  | <0.01 |
| Absent | 99 | 28 | 71 |  |
| Present | 9 | 8 | 1 |  |
| **TNM stage** |  |  |  | 0.378 |
| I and II | 14 | 3 | 11 |  |
| III and IV | 94 | 33 | 61 |  |

*** missing case**
