# Supplementary material for: Circular RNA EIF4G3 suppresses gastric cancer progression through inhibition of β-catenin by promoting δ-catenin ubiquitin degradation and upregulating SIK1
Source: Mol Cancer. 2022 Jul 2;21:141. doi: 10.1186/s12943-022-01606-9 (PMC9250212; doi:10.1186/s12943-022-01606-9)
Supplement: Supplementary file 3 — Additional file 3: [file 12943_2022_1606_MOESM3_ESM.zip › 2-Supplementary Table 3.docx]

**Supplementary Table 3: Primer sequences for qRT-PCR**

| **Name** | **Primer** | **Sequence(5'-3')** |
| --- | --- | --- |
| CircEIF4G3 | Forward | CCTCAAACCCGTTCTCCGAA |
|  | Reverse | CCGTGCTGTAGACTGCTGAG |
| Actin | Forward | CACGAAACTACCTTCAACTCC |
|  | Reverse | CATACTCCTGCTTGCTGATC |
| N-cadherin | Forward | AGTCAACTGCAACCGTGTCT |
|  | Reverse | AGCGTTCCTGTTCCACTCAT |
| Vimentin | Forward | GAGCTGCAGGAGCTGAATG |
|  | Reverse | AGGTCAAGACGTGCCAGAG |
| SIK1 | Forward | CTCCGGGTGGGTTTTTACGAC |
|  | Reverse | CTGCGTTTTGGTGACTCGATG |
| δ-catenin | Forward | ATGTTTGCGAGGAAGCCGC |
|  | Reverse | CGAGTGGTCCCATCATCTG |
| E-cadherin | Forward | CGCATTGCCACATACACTCT |
|  | Reverse | TTGGCTGAGGATGGTGTAAG |
| cyclin D1 | Forward | CCGAGAAGCTGTGCATCTAC |
|  | Reverse | CTTCACATCTGTGGCACAGAG |
