# Supplementary material for: Circular RNA EIF4G3 suppresses gastric cancer progression through inhibition of β-catenin by promoting δ-catenin ubiquitin degradation and upregulating SIK1
Source: Mol Cancer. 2022 Jul 2;21:141. doi: 10.1186/s12943-022-01606-9 (PMC9250212; doi:10.1186/s12943-022-01606-9)
Supplement: Supplementary file 3 — Additional file 3: [file 12943_2022_1606_MOESM3_ESM.zip › Supplementary Table 4.docx]

**Supplementary Table 4: Antibodies used in this study**

| Antigens | Manufacturers | Applications | |
| --- | --- | --- | --- |
| E-cadherin | Cell Signaling Technology, Beverly, MA, USA | | 1:1000 for WB |
| N-cadherin | Cell Signaling Technology, Beverly, MA, USA | | 1:1000 for WB |
| β-catenin | Cell Signaling Technology, Beverly, MA, USA | | 1:1000 for WB |
| Vimentin | Cell Signaling Technology, Beverly, MA, USA | | 1:1000 for WB |
| slug | Cell Signaling Technology, Beverly, MA, USA | | 1:1000 for WB |
| c-Myc | Cell Signaling Technology, Beverly, MA, USA | | 1:1000 for WB |
| cyclin D1 | Cell Signaling Technology, Beverly, MA, USA | | 1:1000 for WB |
| GAPDH | Cell Signaling Technology, Beverly, MA, USA | | 1:2000 for WB |
| SIK1 | Proteintech, Chicago, USA | | 1:500 for WB |
| δ-catenin | Proteintech, Chicago, USA | | 1:500 for WB |
| TRIM25 | Proteintech, Chicago, USA | | 1:1000 for WB |
| Ub | Abcam, Cambridge, MA, USA | | 1:1000 for WB |
